# Artificial Neural Networks: An Innovative Approach Used for Elucidation of Ionization Processes in Supercritical Fluid Chromatography-Mass Spectrometry

**DOI:** 10.1021/acs.analchem.5c00152

**Published:** 2025-05-10

**Authors:** Kateřina Plachká, Veronika Pilařová, Tat′ána Gazárková, Jean-Christophe Garrigues, František Švec, Lucie Nováková

**Affiliations:** † Department of Analytical Chemistry, Faculty of Pharmacy in Hradec Králové, 37740 Charles University, Akademika Heyrovského 1203/8, 500 03 Hradec Králové, Czechia; ‡ SOFTMAT (IMRCP) Laboratory, SMODD Team, 27051 CNRS, Toulouse III Paul Sabatier University, 31400 Toulouse, France

## Abstract

Understanding and predicting mass spectrometry responses in supercritical fluid chromatography-mass spectrometry (SFC-MS) is critical for optimizing detection across diverse analytes and solvent compositions. We present a novel approach using artificial neural networks (ANN) to explore the complex relationships between molecular descriptors of analytes and MS responses in different makeup solvent compositions enabling SFC-MS coupling. 226 molecular descriptors were evaluated for compounds under standardized SFC conditions, with 24 makeup solvent compositions. These makeup solvents included pure alcohols and methanol with varying concentrations of volatile additives. Our results highlight distinct ionization processes for the two most commonly used soft ionization techniques: (i) electrospray ionization (ESI), primarily involving proton or cation transfer, and (ii) atmospheric pressure chemical ionization (APCI), associated with charged ion transfer. Principal component analysis of weights assigned to molecular descriptors reveals that, in positive detection mode, these descriptors effectively differentiate ionization efficiency between ESI and APCI. In contrast, this differentiation is less pronounced in negative mode, where the variance explained is more homogeneously distributed, with stronger discrimination observed when NH_3_ is used as an additive to the organic modifier. These findings provide critical insights into the influence of molecular descriptors and solvent composition on ionization efficiency, serving as a foundation for future investigations into SFC-MS optimization. This proof-of-concept underscores the feasibility of using predictive models to advance understanding of ionization efficiency and offers a valuable framework for refining SFC-MS workflows in analytical chemistry.

## Graphical abstract

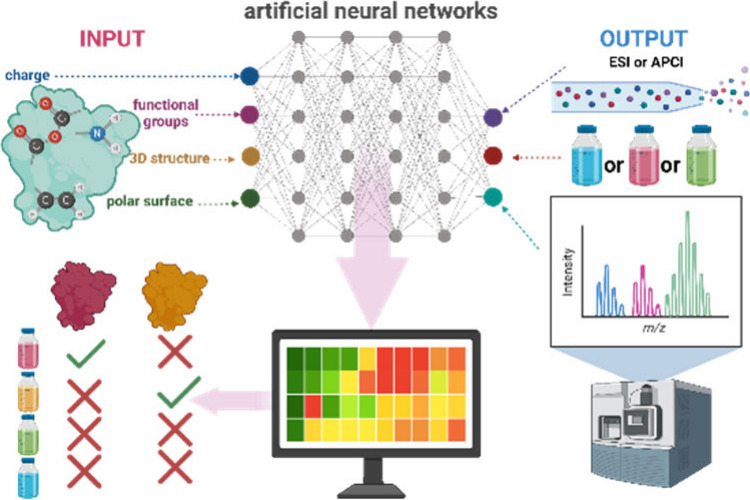

Current packed-column supercritical fluid chromatography (SFC) became very important in many application areas.^1^ An increased interest in SFC stems from the unique physicochemical properties of the mobile phase, which consists of carbon dioxide, organic modifiers, and a small amount of additives.^2–4^ The separation of large spectra of diverse compounds present in varying concentrations in complex matrices requires the hyphenation of SFC with mass spectrometry (MS) to increase sensitivity and selectivity.^5^

Atmospheric pressure ionization sources, adapted from liquid chromatography (LC)-MS, are the gold standard in most SFC-MS applications.^6,7^ Electrospray ionization (ESI) is dominant for the ionization of polar to moderately polar compounds. Ions are formed in the liquid phase through droplets charged by high voltage applied to a capillary tip, their evaporation, and Coulomb fission. Atmospheric pressure chemical ionization (APCI) is used to ionize small nonpolar molecules.^6,7^ In the APCI, the process is carried out in a gas phase. The solvent molecules are ionized via a corona discharge and then interact with the analyte molecules via ion-molecular reactions.^7^

While both SFC-MS and LC-MS utilize the same ionization sources, some differences in ionization mechanisms have been noted due to the nature of the SFC mobile phase. Thite et al.^8^ observed and described three possible mechanisms contributing to ionization in SFC-MS: thermospray ionization, charge residue ionization, and sonic spray ionization. Additionally, an unexpected ionization behavior was observed when no high voltage was applied in the ion source, including ESI and APCI, suggesting the possibility of preformed ions in the solution. Several studies have suggested that alkoxyl carbonate ions are formed when CO_2_ is mixed with an alcohol modifier. This hypothesis was experimentally confirmed by the pH decrease in the CO_2_/methanol mobile phase^9^ and by confirmation of ions corresponding to alkoxyl carbonic acids in the effluent reaching the MS for ESI^10^ and APCI.^11,12^ Fujito et al.^10^ reported that alkoxyl carbonic acid contributed to ion generation as an H^+^ donor in ESI^+^. An increased sensitivity was observed as the proportion of CO_2_ in the CO_2_/methanol mixture increased.^10^ In ESI^−^, a decrease in MS response was observed when CO_2_ was added to methanol as the acid formed interfered with the ionization of analytes.^10,11^ In APCI, the CO_2_ is involved in ion-molecule equilibria in the corona plasma discharge. Therefore, the CO_2_/methanol ratio did not have a straightforward effect on the sensitivity.^11^ Nevertheless, the results indicated the significant role of CO_2_ as a precursor for the formation of reaction species involved in the ionization.^12^

The presence of CO_2_ in the mobile phase is also the origin of the challenges of SFC-MS hyphenation, including (i) the decompression cooling effect, (ii) possible analyte precipitation in the capillary prior to the MS as a consequence of the pressure drop, density decrease, and evaporation of CO_2_, and (iii) poor ionization of analytes affected by a high proportion of CO_2_ in the mobile phase. Therefore, several specially designed interfaces are used to enable SFC-MS hyphenation.^13–15^ The two preferred interfaces use the sheath pump, which provides the additional post-column makeup solvent that affects the ionization efficiency.^13,16,17^ Makeup solvent typically consists of methanol with or without volatile additives, such as organic acids, e.g., formic acid, salts, e.g., ammonium formate, bases, e.g., ammonia, and/or water. The additives further support the ionization of the analytes without interfering with the separation. However, they can also compete with analytes for a charge and suppress ionization. Water content can also decrease the response due to the higher surface tension of water. A higher additive concentration is required for signal enhancement in APCI.^18,19^ Hence, the composition of the makeup solvent must be carefully optimized for each application, as it is strongly dependent on the physicochemical properties of the analytes and SFC-MS conditions.^6,10,19–22^

No general conclusions have been drawn about the makeup solvent composition in relation to the ionization source and the physicochemical properties of analytes so far. In our previous work, we proposed the regression equations that describe the correlations and dependencies of some physicochemical properties of analytes and SFC-ESI-MS response.^23^ Nonetheless, a more comprehensive characterization of the analyte properties is required to fully understand the ionization processes. Bieber et al.^24^ proposed the prediction of ESI efficiency for SFC-MS using a pre-trained model, molecular descriptors, and artificial neural networks. Their study investigated the possibility of quantification without any standard using a constant makeup solvent composition throughout the whole study, thus omitting the investigation of its effect.^24^ To date, no study has focused on describing ionization processes in SFC-APCI-MS.

The aim of this comprehensive study was to investigate the effect of the makeup solvent composition, including the different organic solvents and additives in a wide range of concentrations, using two ionization sources, ESI and APCI. The set of 95 compounds with different physicochemical properties described by 226 molecular descriptors was analyzed and evaluated. The results promise to simplify the optimization of the SFC-MS method as we determined molecular descriptors responsible for increased MS response using each of the tested makeup solvents. By matching the solvent composition with analyte properties, ionization efficiency in the MS can be maximized, thereby improving detection limits. The *in silico* optimization of the makeup solvent composition also reduces the volume of organic solvents used, promoting the environmental friendliness of the SFC technique while also improving the accuracy and robustness.

## Experimental Section

### Materials and Chemicals

The reference standards are listed in the SI 1 Table S1. Standards were purchased from Sigma-Aldrich (Germany) or kindly donated by Zentiva, k.s. (Czechia). All standards were dissolved in acetonitrile (ACN), methanol (MeOH), tetrahydrofuran (THF), or MeOH/ACN (1/1) (SI 1 Table S1) to obtain 1 mg/mL. Pressurized liquid CO_2_ 4.5 grade (99.9995%) was purchased from Messer (Czechia). MeOH, ethanol (EtOH), isopropanol (IpOH), ACN, and water in LC/MS grade quality were provided by VWR International (Germany). Ammonia 4 mol/L solution in methanol for LC/MS and LC/MS grade ammonium acetate (≥99.99%) were purchased from Sigma-Aldrich (Germany). Formic acid for LC/MS (≥99%) was purchased from VWR International (Germany). Acetic acid (100%) and ammonium formate (≥99.0%) in LC/MS grade were purchased from Merck (Germany).

### Analytical Instrumentation and Conditions

The experiments were carried out on an Acquity UPC^2^ (Waters, USA) supercritical fluid chromatography system equipped with a binary pump, an autosampler, a column thermostat, a back pressure regulator (BPR), and a PDA detector. The Xevo TQ-XS triple quadrupole mass spectrometer (Waters) was coupled via a commercial SFC-MS dedicated pre-BPR splitter device with an additional isocratic pump to deliver the makeup solvent (Waters). The system was operated by MassLynx V4.2 software. The chromatographic conditions are listed in SI 1. The makeup solvent (Table 1) flow rate was set up at 0.3 mL/min. Analyses were carried out using ESI and APCI (Waters). Positive and negative ionization modes were used for the analyses with selected ion monitoring (SIM) of molecular ions corresponding to [M + H]^+^ and [M – H]^−^. Ionization source parameters for ESI: capillary voltage 2.0 kV, ion source temperature 150 °C, desolvation temperature 500 °C, desolvation gas flow 1000 L/h, cone gas flow 150 L/h, and nebulizer pressure 5.0 bar. For APCI: corona current 2.0 kV, ion source temperature 150 °C, probe temperature 550 °C, desolvation gas flow 1000 L/h, cone gas flow 150 L/h, and nebulizer pressure 5.0 bar.

**Table 1:** List of Tested Makeup Solvents for Individual Ionization Sources^a^

	**ESI**	**APCI**
**Alcohol**	MeOH, EtOH, IpOH	MeOH, EtOH, IpOH
**MeOH + H_2_O**	1; 5; 10; 20 mM	1; 5; 10; 20 mM
	1; 5%	1; 5%
**MeOH + NH_3_**	1; 5; 10; 20 mM	1; 5; 10; 20; 50;
		100 mM
**MeOH + FA**	1; 5; 10; 20 mM	1; 5; 10; 20; 50;
		100 mM
	0.1%	0.1; 0.5%
**MeOH + AA**	1; 10 mM	10; 50; 100 mM
**MeOH + AmF**	1; 10 mM	10; 50; 100 mM
**MeOH + AmAc**	1; 10 mM	10; 50; 100 mM

a FA – formic acid, AA – acetic acid, AmF – ammonium formate, AmAc – ammonium acetate, MeOH – methanol, EtOH – ethanol, IpOH – isopropanol.

### Design of the Study

The study design was adapted from Plachka et al.^23^ Two diol columns (Torus Diol, 100 × 3.0 mm, 1.7 μm, Waters) were used in the study, one dedicated to each organic modifier. Each of the makeup solvents tested was analyzed with both mobile phase compositions within one day. The MS response was always correlated to the quality control (QC) samples that were analyzed immediately prior to the makeup solvent tested in order to reduce the inter-day variability of MS responses. The QC samples corresponded to the same analytes but were measured using 10 mmol/L NH_3_ in MeOH as a makeup solvent.

### Data Processing and Evaluation

The initial data processing and evaluation were adapted from Plachka et al.^23^ followed by a detailed description and characterization of the analytes by molecular descriptors and training of artificial neural networks (ANN). The raw data were processed using TargetLynx XS software (Waters) and evaluated in MS Excel. The MS responses were corrected by the respective QC samples. The MS responses of the first measured QC samples were considered as 100%. Then, the responses of the QC samples measured throughout the study were correlated to this 100%, and the factor obtained was used to correct the responses of the analytes measured after each QC.The measured-adjusted responses were then calculated according to published literature (SI 1 Equation S1).^23,25–27^

The 3D structures of all analytes were optimized by semi-empirical AM1 quantum mechanical calculations (MOPAC application of the Chem 3D Pro version 14.0 software, CambridgeSoft). A root mean square (RMS) gradient of 0.100 was used to minimize energy for compounds. These optimized structures were then used to calculate 2D and 3D molecular descriptors (CDK Descriptor Calculator, v.1.4.8). The 226 molecular descriptors (SI 1 Table S2) included topological, constitutional, hybrid, electronic, and geometric descriptors related to the 2D and 3D structure of the molecule, its surface area, moment of inertia, fragment counts, complexity, chi clusters, chi chains, chi pathways, and charge. All the molecular descriptors and MS responses were normalized by dividing by the maximum value. ANNs were created using the neural network simulator in Matlab R2023a with the deep learning toolbox V.23.2 (The MathWorks, Inc., USA) with a sigmoid activation function, a backpropagation learning algorithm with 1500 learning cycles to identify key molecular descriptors linking the structure of analytes to their ionization under different conditions. All 226 molecular descriptors, i.e., input neurons, were connected to the output neurons, i.e., MS response. The ANN training process employed a backpropagation algorithm with multiple termination criteria to ensure optimal network performance and reliability. Training was halted when reaching the maximum number of training epochs, surpassing the predefined time limit, attaining the target performance threshold, and a performance gradient dropping below the target threshold. To evaluate the effectiveness of regression learning, only ANN models with a Root Mean Squared Error (RMSE) below 0.5 between the target training values and the predicted outputs after 1500 learning cycles were retained. Following the learning phase, the assigned weights for each input neuron were extracted. The higher the weight assigned by the ANN, the more the descriptor affects the ionization.^28^ Molecular descriptors with absolute ANN-assigned weights exceeding 1.5 were identified as key descriptors, and all descriptors were ranked according to these absolute weight values. The molecular descriptor with the highest absolute weight was assigned a rank of 1 with the molecular descriptor with the lowest absolute weight ranked as 226. The changes in rankings were then used to describe changes in the ionization processes.

Principal component analysis (PCA) was used to reduce the dimensionality of the dataset by identifying key patterns within the molecular descriptors and to highlight the major sources of variation affecting the MS response based on makeup solvent composition (Matlab R2023a).

## Results and Discussion

Several studies have investigated the specifics of ionization in SFC-MS with the presence of CO_2_, but no general conclusions have been drawn. Thus, the optimization of SFC-MS methods remains a challenging and laborious experimental endeavor. This is especially true for the optimization of the makeup solvent composition, which is usually made by testing different solvents one at a time, without a deeper understanding of the makeup solvent effect on ionization and MS response. This proof-of-concept study was designed to mimic the commonly used conditions as closely as possible. Thus, it simulates the best-case scenario, i.e., perfect separation of target analytes using gradient elution, analysis of pre-treated samples without matrix interferences, and optimized ionization source parameters. The testing of coelution and matrix interferences is the subject of future studies. The same ionization source parameters were used throughout the study with all tested makeup solvents to focus directly on the differences caused by the makeup solvent composition. Further investigation is needed to elucidate the relationship between makeup solvent composition and differences in optimal settings of the ionization source.

The use of ANN offers unparalleled capacity, allowing the definition of correlations in large data sets based on rigorous rules set for the training cycles. We have applied the power of ANN to elucidate the processes that occur during ionization in ESI and APCI when different makeup solvents are used. Several parameters affect the ionization efficiency in SFC-MS, including the SFC conditions, the splitting ratio in the SFC-MS interface, the ionization source, makeup solvent composition, and analyte properties. The generic gradient and SFC conditions in this study were used to obtain as general results as possible. The effect of the splitting ratio in the SFC-MS interface was compensated by adjusting the obtained MS responses to 100 μL of eluent entering the MS. The use of ANN enabled us to describe in detail the effect of the remaining three parameters, i.e., (i) the effect of the type of ionization source when using (ii) different makeup solvent compositions to analyze (iii) compounds with different physicochemical properties of analytes described by 226 molecular descriptors. The well-trained ANN determined which molecular descriptors play a crucial role in ionization processes of ESI and APCI and how their effect changes based on the makeup solvent composition.

Based on the molecular descriptors of detected analytes adjusted by ANN-assigned weights, the tested ionization sources clustered separately in the positive ionization mode with a closer correlation in the negative ionization mode in PCA. Figure 1 shows that, in positive ionization mode, the weights assigned to the molecular descriptors of the analytes help explain the variance in ionization efficiency differentiated between ESI and APCI, particularly for the first principal component (PC1). In negative mode, this discrimination is less distinct. PC1, which accounts for 46% of the variance (b) and 40% (d), displays a more homogeneous distribution of weights assigned to different analytes, especially when MeOH is used as organic modifier. In the presence of NH_3_ in the organic modifier (d), the separation between ESI and APCI modes is more pronounced but with less discrimination than that observed in positive mode.

**Figure 1:**
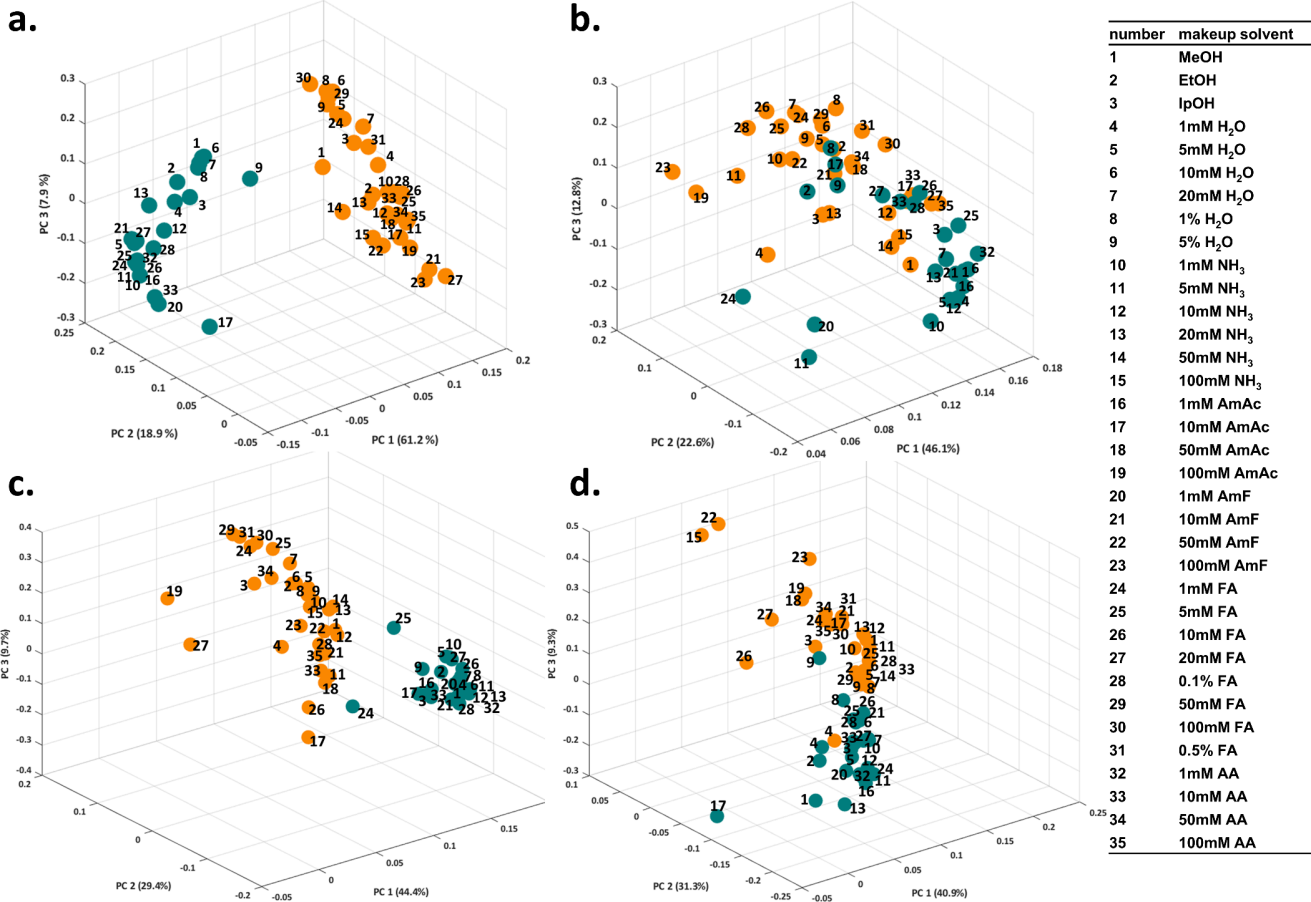
Multivariate principal component analysis of ANN-assigned weights of the molecular descriptors based on makeup solvent composition and ionization source used: ESI - green, APCI - orange. The same SFC-MS conditions were used for all analyses and MeOH as an organic modifier in positive (a) and negative (b) ionization mode, and 10 mmol/L NH_3_ in MeOH as an organic modifier in positive (c) and negative (d) ionization mode.

APCI is associated with charged ion transfer, whereas ESI primarily involves proton or cation transfer. Figure 1 indicates that, in positive ion mode, the ions driving ionization differ sufficiently to allow the underlying mechanisms to be elucidated using molecular descriptors. In contrast, in negative ion mode, the ions involved are either distinct or exhibit significant similarity. These observations underscore the need for a more detailed molecular investigation to further explore these ionization mechanisms. It is important to note that the number of ionized and detected compounds in negative mode differed by approx. 15% (SI 1 Table S1), which may influence the observed variance. This is largely attributed to the chemical structures of the compounds analyzed. While it is possible to select compounds better suited to negative mode ionization, the set of analytes used in this study was purposefully constructed from bioactive compounds commonly analyzed by SFC-MS, ensuring relevance and alignment with real-life applications.

For MeOH as an organic modifier in negative ionization mode, the ionization processes in ESI and APCI were more closely related (Figure 1b), and an even greater effect of makeup solvent composition on ionization was visible. The use of ammonia in organic modifier affected the ionization in both ionization sources. ESI and APCI clustered closer in the positive ionization mode (Figure 1c). Furthermore, the differences in ionization caused by makeup solvents were minimized in ESI, as most makeup solvents clustered close together. Greater variation due to makeup solvent composition was observed in negative ionization mode (Figure 1d). However, the more pronounced similarity between ESI and APCI was again observed. The PCA analysis confirmed that different properties of analytes are responsible for increased and decreased ionization efficiency in ESI and APCI, respectively, especially when using different makeup solvent compositions. Therefore, we examined the molecular descriptors with the highest ANN-assigned weights and used them to elucidate the differences in ionization.

The reader is referred to SI 1 Table S2 for an explanation of all molecular descriptors discussed in this paper. The original papers provide a more detailed explanation of the theory behind particular molecular descriptors and their calculations.^29–39^ Results and discussion focusing on negative ionization mode can be found in Supporting Information 2.

### Ionization in SFC-MS Using Methanol As Organic Modifier in Positive Ionization Mode

#### Methanol as Makeup Solvent

Figure 2 shows the key molecular descriptors affecting the ionization efficiency by the highest weight for each ionization source and makeup solvent. Overall, these molecular descriptors can be divided into several groups as clustered in gray. The ESI^+^ (Figure 2a) was strongly affected by the (i) charged surface area of the analyte. The molecular descriptors related to the surface area with a negative partial charge, such as PNSA-3 and WNSA-3 (SI 1 Table S2), were assigned highly negative weights by ANN. Both descriptors are based on the charge-weighted partial negative surface area of the molecule, i.e., compounds with high negative partial charge are assigned mathematically low values of PNSA-3 and WNSA-3. The negative ANN-assigned weight indicates that molecules with high PNSA-3 and WNSA-3 have lower ionization in ESI^+^, corresponding to a high affinity of the negatively charged surface area for protons. In contrast, high values of relative positive charge surface area (RPCS) and charge-weighted partial positive surface area (PPSA-3) had a negative effect on ionization. However, the overall charge state of the molecule has to be considered in ESI^+^, as shown by the positive weights of PPSA-1 (sum of surface areas on positive parts of the molecule) and DPSA-1 and DPSA-3 (differences between surface area with positive and negative partial charges). They show that although the negative charge was preferred, it should be localized on a small fraction of the surface area of the molecule, since compounds with predominantly positive partial charge surface area have significantly higher ionization efficiency in ESI^+^. These molecular descriptors showed the opposite behavior in APCI^+^ (Figure 2b). Charged surface area affected the ionization efficiency to a lesser extent in APCI^+^. Furthermore, compounds with high relative negative charge surface area (RNCS) and high values of PNSA-3 and WNSA-3 had lower ionization efficiencies compared to compounds with high RPCS and FPSA-3. The importance of the polar surface area for APCI^+^ was further demonstrated with the tpsaEfficiency descriptor calculated as a topological polar surface area/molecular weight. This suggests an intermolecular charge transfer^40–42^ where the protons from the methoxycarbonyl acid formed in the mobile phase are attracted to the analyte with a partially negatively charged surface.

**Figure 2:**
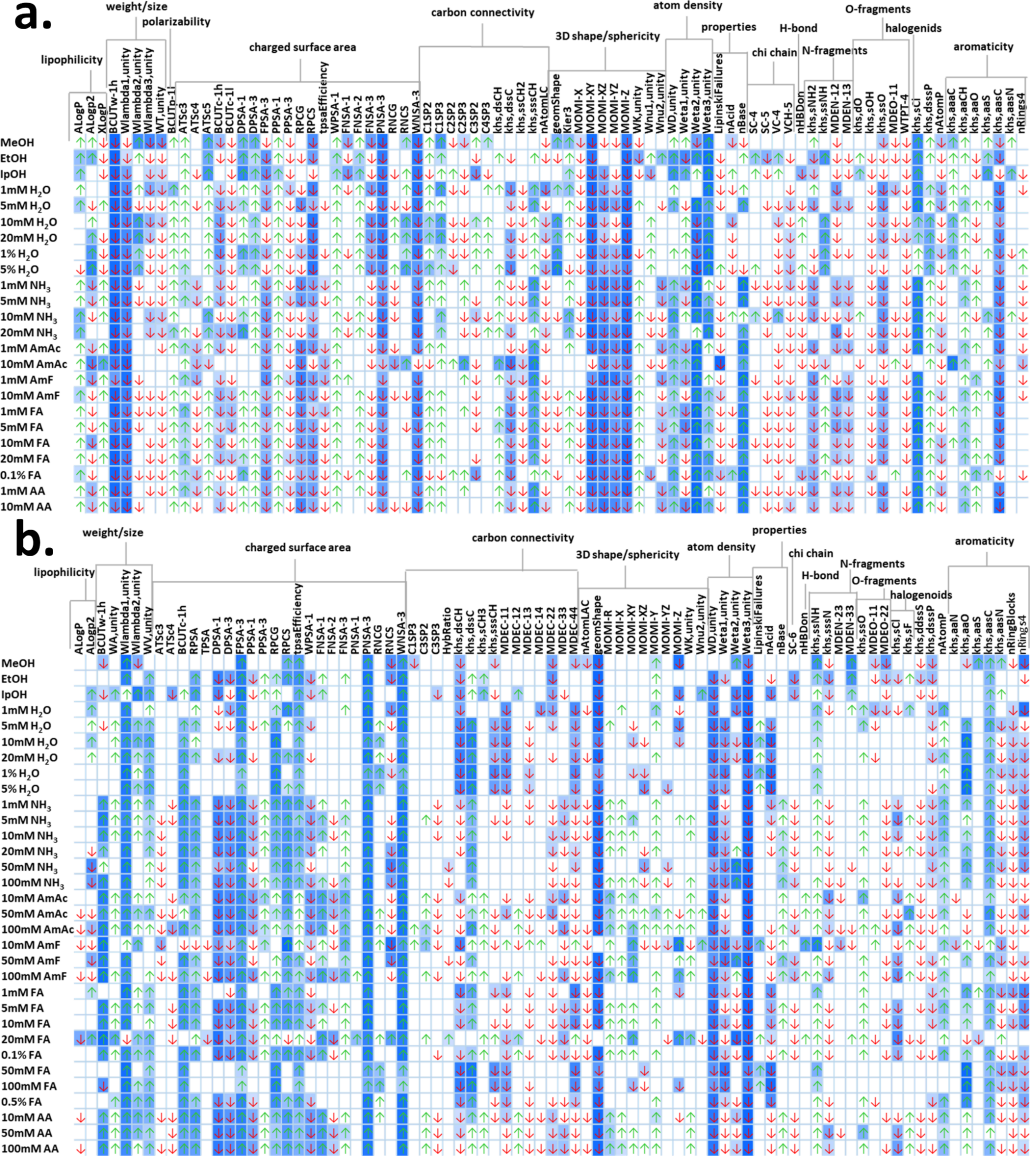
Key molecular descriptors affecting ionization in ESI^+^ (a) and APCI^+^ (b) when using MeOH as organic modifier. The shade of blue in the heatmaps corresponds with a ranking of the molecular descriptor for each makeup solvent composition (ranking 1 = the highest absolute ANN-assigned weight = the darkest blue). ↑ - increasing and ↓ - decreasing effect on ionization.

(ii) The second set of molecular descriptors was related to carbon connectivity and the presence of heteroatoms. The MS response in APCI^+^ was negatively affected by the increasing distance edge between secondary (MDEO-22) and primary (MDEO-11) oxygens. However, covalently bonded oxygens are beneficial contrary to aromatically bonded oxygens (khs.aaO). Similarly, the presence of tertiary amines decreased ionization efficiency in APCI^+^ (negative weight of khs.sssN and the positive weight of MDEN-33), while secondary amines (khs.ssNH) increased ionization. Khs.dsCH counts R1=CH–R2 parts of the molecule, where R can be any heteroatom and/or carbon. Since the ANN-assigned weight of MDEO-22 suggests a preference for the presence of oxygens in the molecule, we can state that carbon fragments, especially CH_2_=CH–R (khs.dsCH), −CH_3_ (C1SP3), and −CH<, had a negative effect on the ionization efficiency in APCI^+^. In ESI^+^, a strong positive effect of chlorine substitutions (khs.sCl) was observed. The presence of primary amines increased ionization (positive weight of khs.sNH2 and negative weight of MDEN-12). In contrast to APCI^+^, secondary oxygens (khs.ssO) decreased ionization in ESI^+^, while primary oxygens were beneficial (negative weight of MDEO-11). Overall, functional groups with acidic and basic properties had a small but negative effect on ionization in ESI^+^, whereas they did not significantly affect APCI^+^. The hybridization state of the carbons in the molecule also played an important role in ionization in ESI^+^. While fragments with −CH_2_−, =C=, and aromatic carbons with additional covalent bonds (C2SP3, C2SP2, khs.aasC) decreased ionization, carbons with two/three aromatic bonds (khs.aaaC, khs.aaCH), =CH_2_, −CH_3_, >C=, and >C< groups, had positive effect on ESI^+^.

(iii) Weighted holistic invariant molecular (WHIM) descriptors are related to the information about 3D properties related to size (Wlamda, Wλ), shape (Wnu, Wν), symmetry, and atom distribution (Weta, Wη) of the molecule weighted by different schemes.^34^ For each weighting scheme, a set of statistical indices is calculated on the atoms projected onto each of the three PC.^34^ Thus, the opposite behavior of Weta2.unity and Weta3.unity (Figure 2a) corresponds to different atom distributions along the PC axis. The same applies to Wlambda1, Wlambda2, and Wlambda3 related to molecular size, which showed strong but varying effects on ionization with both sources. The key effect of molecular size for ESI^+^ was further demonstrated with the BCUTw-1h parameter. BCUT is based on a weighted version of the Burden matrix accounting for both the connectivity and the atomic properties with atomic weight (BCUTw), partial charge (BCUTc), and polarizability (BCUTp) weighting schemes, where the highest (−1h) and lowest (−1l) eigenvalues can be calculated.^38^ BCUTw-1h significantly decreased MS responses in both APCI^+^ and especially ESI^+^.

Finally, (iv) the 3D shape of the molecule can be described by the moment of inertia (MOMI), which is calculated using three perpendicular axes passing through the center of mass and the mass distribution from these axes (SI 1 Figure S1). Three descriptors and their ratios can be calculated that describe four types of 3D shapes of molecules: linear, symmetric top, spherical, and asymmetric top. High values of MOMI had a strong negative effect on the ionization in ESI^+^. Only MOMI-Z and MOMI-Y affected ionization in APCI^+^, where molecules with low values of MOMI-Z and high values of MOMI-Y, i.e., linear and symmetric top molecules, were more easily ionized (Figure 2b). The 3D shape of the molecule can also be described by geomShape (circular/spherical compounds = 1, linear molecules = 0). Spherical compounds had increased MS responses in ESI^+^, in contrast to the negative effect of sphericity in APCI^+^.

#### The Effect of Alcohol Type

MeOH is by far the most used solvent in the makeup solvent composition.^6^ However, any organic solvent miscible with CO_2_ can be used. The use of EtOH had an ambiguous effect on the MS response in ESI^+^ and APCI^+^, with increased ionization for 23% and 32% of compounds and decreased ionization for 56% and 50% of compounds, respectively (SI 1 Figure S2). IpOH resulted in a significant increase in MS response for >80% of compounds in both ESI^+^ and APCI^+^. This study was designed to replicate real-life scenarios where the ionization source parameters are typically optimized first, followed by optimization of makeup solvent composition. Hence, the same ionization source parameters were used for all experiments. The alcohols differ in their properties, such as surface tension, which affects their droplet formation and evaporation efficiency. Thus, the used ionization parameters might not be optimal for all alcohols, as higher or lower flow rates of desolvation gas could be beneficial. Thus, this comparison of ionization efficiencies applies, but further investigation of the relationship between makeup solvent composition and ionization source parameters is needed.

The changes in molecular descriptor weights were compared to describe the changes occurring in the ionization efficiency when EtOH and IpOH are used (SI 1 Figure S3a). To elucidate these variations using ANN, we must keep in mind that ANN assigned the weights to molecular descriptors always within one set of experimental data. Thus, it is necessary to evaluate also the ranking of the molecular descriptors within each set (Figure 2a). In ESI^+^, the ionization was more strongly affected by the number of basic (nBase) and acidic (nAcid) groups with EtOH and IpOH, respectively. The number of H-bond donors had a strongly negative effect on the ionization efficiency with IpOH (Figure 2a). Otherwise, most of the changes occurred in descriptors related to the valence and simple clusters (VC-4, SC-4, SC-5) and WHIM (Weta2, Weta1, WD, Wlambda2). The decreasing effect of nAcid was stronger with EtOH and IpOH than MeOH in APCI^+^ (Figure 2b). Molecules with predominantly positive partial charged surface area (DPSA-1, DPSA-3, WPSA-1) were less efficiently ionized using EtOH and IpOH. The positive effect of WHIM descriptors related to molecular size (WA, Wlambda) became more pronounced.

#### The Effect of Additive

The use of additives in makeup solvents, typically at concentrations around 10 mmol/L, is common in SFC-MS. However, such concentration can result in ionization suppression due to competition for charge. To elucidate the effect of additives on the ionization, we described their effect at low concentration and then evaluated the effect of increasing concentration. In our study, we aimed only at the effect of additives in methanolic makeup solvent, which is preferred in most studies.^6^ Nevertheless, its effect might differ when using different makeup solvents, e.g., ethanol and isopropanol.

Comparison of MS responses shows changes in the ionization efficiency and processes due to the additive in makeup solvent, as confirmed by the SD in the weights assigned to the corresponding molecular descriptors (SI 1 Figure S4a).

#### Water

1 mmol/L H_2_O in MeOH (≈0.02%) is a much lower concentration than typically used. Nevertheless, even this low concentration affected MS responses, especially in APCI (Figure 3a), with decreased MS responses for >80% of the compounds. Responses in ESI^+^ were affected to a lesser extent but also predominantly decreased. All molecular descriptors were assigned similar weights using both MeOH and 1 mmol/L H_2_O in MeOH, with SD usually <1 (SI 1 Figure S4a). Thus, the ranking of the molecular descriptors was compared next with rankings obtained using pure MeOH (Figure 3b,c) as the makeup solvent, which confirmed smaller effect of water in ESI^+^ (R^2^ of 0.64) vs APCI^+^ (R^2^ of 0.47). The influence of the WHIM descriptors Weta1 and Weta2 decreased in ESI^+^ (Figure 2a), as did the effect of the negative partial charge present on the surface area (PNSA-1) and the topological shape. Conversely, the positive effect of the methine group khs.sssCH (>C−) and khs.dsCH (=C−) became more pronounced. In APCI^+^, the effect of oxygen in the molecule (khs.aaO, khs.ssO), molecular dimensions (WA, unity), and AlogP2 increased ionization by a greater extent, whereas molecular density (WD.unity), large molecular distance edge between methyl groups (−CH_3_, MDEC-11, MDEC-14), and positive surface area (DPSA-3) had a more significant decreasing effect.

**Figure 3:**
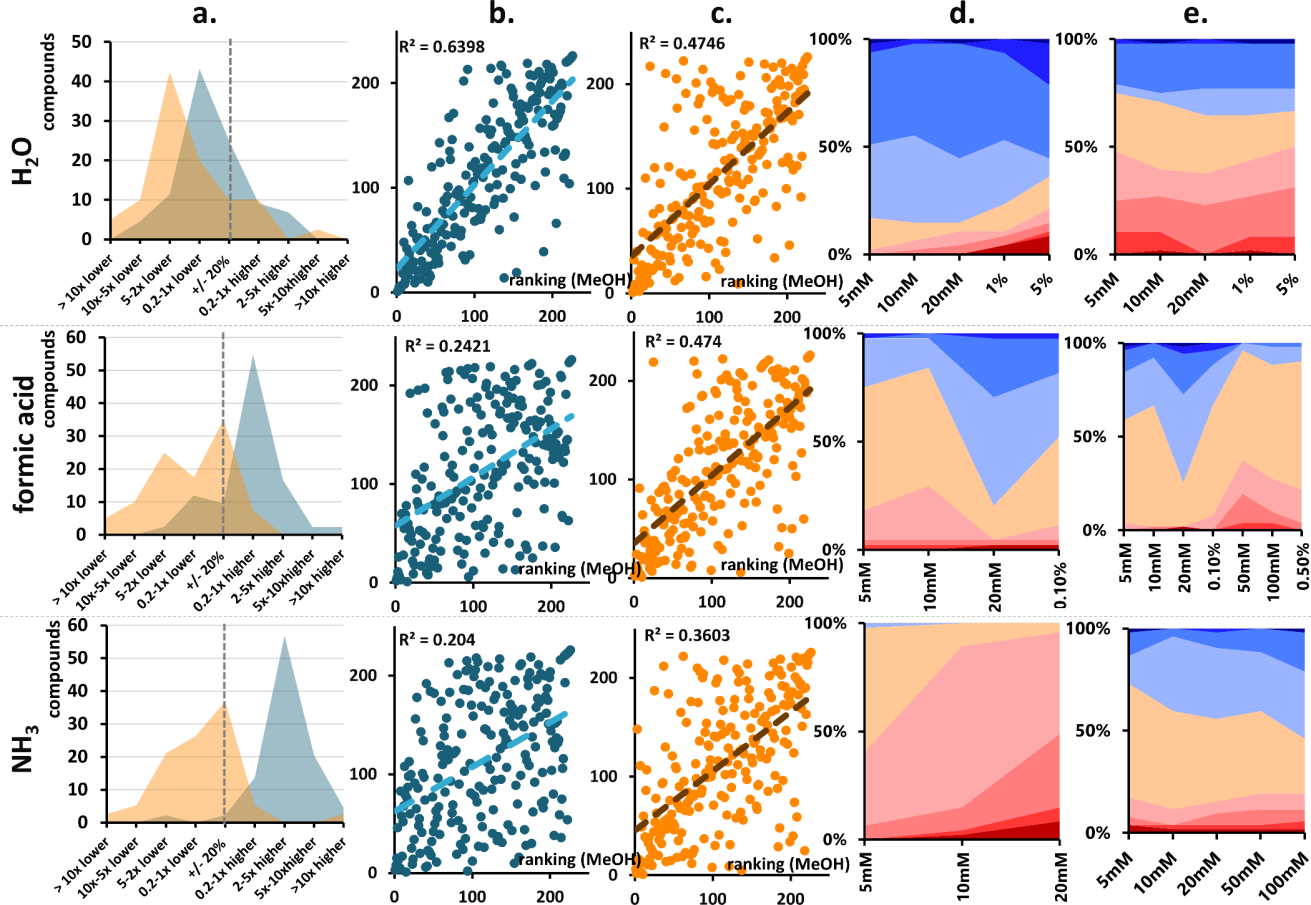
Effect of 1 mmol/L additive in makeup solvent on MS responses compared to MeOH (a) and comparison of rankings of molecular descriptors (b, c) for ESI^+^ (blue) and APCI^+^ (orange). Effect of increasing additive concentration on MS responses in ESI^+^ (d) and APCI^+^ (e) compared to MS responses obtained using 1 mmol/L additive in MeOH. Over 10-fold lower (dark red), 10–5-fold lower (red), 5–2-fold lower (dark pink), 0.2–1-fold lower (pink), within +/– 20% (orange), 0.2–1-fold higher (the lightest blue), 2–5-fold higher (light blue), 5–10-fold higher (blue), over 10-fold higher (dark blue). All results were obtained using MeOH as the organic modifier.

The increasing concentration of H_2_O (Figure 3d,e) had a positive effect on the ionization of >80% of the compounds in ESI^+^, while both a strong increase and decrease were observed in APCI^+^ based on the physicochemical properties of analytes. The increasing concentration of H_2_O mainly affected the weights of molecular descriptors related to lipophilicity and molecular weight (Figure 2). High values of ALogP2 are assigned to highly lipophilic compounds, similar to logP. However, additional corrections for molecular topology and specific interactions are accounted for including topological features (e.g., branching, rings, and proximity of polar groups), which can reduce or amplify the lipophilicity compared to logP, and intramolecular hydrogen bonding, which can increase the apparent lipophilicity, giving a higher AlogP2 value for molecules that are otherwise hydrophilic by nature. Compounds with high values of AlogP2 had increased MS responses using ESI^+^_,_ with a further increase with increasing concentration of H_2_O. BCUTw-1h showed the opposite behavior, indicating that compounds with higher molecular weights will have decreasing MS responses with higher concentration of H_2_O. The combination of AlogP2 and BCUTw-1h behaviors suggests that highly hydrophilic compounds with lower molecular weight will be more easily ionized using 5% H_2_O while lower ionization efficiency will be observed for high molecular weight lipophilic compounds. The change from positive to negative weights of BCUTw-1h with increasing concentration of H_2_O observed for APCI^+^ means that higher molecular weight compounds ionized more easily using 1 mmol/L H_2_O.

Other molecular properties strongly affected by the increasing concentration of H_2_O were related to charge state and 3D shape of the compound. Higher concentration of H_2_O increased the positive effect of the relative negative charge surface area (RNCS) on ionization in ESI^+^. The opposite behavior was observed in APCI^+^, with positive weights assigned to PNSA-3 and WNSA-3 corresponding to increased ionization efficiency for compounds with the lowest negative charge. The positive effect of Cl- groups on ionization in ESI^+^ decreased with increasing H_2_O concentration. The positive effect of the geomShape increased with increasing H_2_O concentration in ESI^+^, which can be related to more stable drop formation and higher ion density distribution. Ion density distributions around a charged object in solution depend significantly on whether the object is a sphere, rod, or plane.^43^ Electrical free energies increase with sphericity, but the effective charge and surface electrical potential decrease with increasing radius of gyration of the spheroid.^43^ High electrical free energies of spheroids in ESI^+^ can be correlated with easy ion pairing with H_3_O^+^ ions from the makeup solvent, resulting in easier ionization. On the other hand, a non-spherical shape was beneficial for APCI^+^.

#### Acids

The use of 1 mmol/L formic acid (FA, 0.004%) mainly increased ionization in ESI^+^, whereas mostly decreased ionization in APCI^+^ (Figure 3a). Based on the violin plots, the largest differences in molecular descriptor weights were observed in ESI^+^ (SI 1 Figure S4a), as confirmed by the ranking comparison. The addition of FA to the makeup solvent altered the ionization behavior to a greater extent in ESI^+^ with R^2^ of 0.2 compared to R^2^ of 0.5 for APCI^+^ (Figure 3b,c).

In ESI^+^, the use of 1 mmol/L FA was beneficial for linear compounds (negative weights of MOMI-XZ and MOMI-YZ and positive weight of Wlambda1.unity). The effect of charge was less pronounced (DPSA-3, FNSA-3, FNSA-1). However, the molecules with high total positive charge and low values of the most positive charge, i.e., with high negative weight of RPCG, were ionized more easily. This was confirmed by the high positive weight of nBase, showing that a higher number of basic functional groups in the molecule is beneficial for ionization. 1 mmol/L FA improved the ionization of compounds with a charged surface (BCUTc-1h), especially with a positive charge (RPCG) in APCI^+^. Compounds with acidic functional groups (nAcid) and high distribution of atoms along the first and third PC axes (Weta1, Weta3) were ionized with lower efficiency than with MeOH.

Overall, an increase in MS response was observed for most compounds in ESI^+^ using higher concentrations of FA (Figure 3d), but at most 2-fold. This corresponds to the similar weights assigned to the molecular descriptors with all tested FA concentrations, which correlated with R^2^ > 0.9 except for 0.1% FA with R^2^ ≈ 0.8 (SI 1 Table S3). Increasing FA concentration increased the negative effect of molecular weight (BCUTw-1h). Furthermore, the presence of acidic functional groups had a positive effect on ionization with 0.1% FA, whereas this molecular descriptor was negligible at lower FA concentrations. Acetic acid as a makeup solvent behaved similarly to FA, as confirmed by R^2^ > 0.9 of ANN-assigned weights between 1 and 10 mmol/L acetic acid (AA) and 1 mmol/L FA (SI 1 Table S3). The molecular descriptors with the highest SD between AA and FA suggest a beneficial effect of 1 mmol/L AA on the ionization of compounds with quaternary carbons (khs.dssC, khs.aasC) and spherical molecules (MOMI-Z, geomShape) with higher molecular weight (BCUTw-1h) when using 10 mmol/L AA.

In APCI^+^, the concentration effect was negligible in most cases. However, several compounds showed 1000-fold higher responses at higher FA concentrations than 1 mmol/L (Figure 3e). Similar to ESI^+^, comparable ionization behavior was observed in APCI^+^ with different concentrations of FA (R^2^ > 0.8), except for 20 mmol/L, where the molecular descriptor weights did not correlate (R^2^ of 0.1). Most of the molecular descriptor weights showed a U-shaped behavior, increasing from 1 to 20 mmol/L and subsequently decreasing. BCUTw-1h, MOMI-Z, geomShape, and negative surface area descriptors PNSA-3 and WNSA-3 negatively affected ionization with 1 mmol/L FA. Their effect became increasingly positive with up to 20 mmol/L FA, and negative again when using 0.5% FA. An opposite trend was observed for positive surface area (DPSA-1). For AA, the weights of BCUTw-1h increased almost linearly with increasing concentration, while weights of PNSA-3, WNSA-3, DPSA-1, MOMI-Z, and geomShape remained similar.

#### Ammonia

Makeup solvent with 1 mmol/L NH_3_ increased MS responses in ESI^+^ for >90% of the analytes (Figure 3a). Mostly unchanged and/or slightly decreased ionization efficiency was observed in APCI^+^. This is consistent with the violin plots (SI 1 Figure S4) showing the highest SD for ESI^+^. Furthermore, the correlation of the molecular descriptor rankings was 0.4 for APCI compared to 0.2 for ESI.

1 mmol/L NH_3_ decreased the negative effect of the negatively charged surface area (FNSA-1), while RPCG became one of the most important molecular descriptors. Compounds with a small total positively charged surface area were ionized to a lesser extent. High positive values of nBase confirmed the benefit of a high positive charge. 1 mmol/L NH_3_ further affected the evaporation and droplet formation in ESI, as shown by the WHIM descriptors related to the molecular shape and distribution (Weta, Wlambda, WT, Wnu) and descriptors related to the 3D shape (geomShape, MOMI). More linear molecules were preferentially ionized using 1 mmol/L NH_3_. In APCI^+^, the compounds with a high most-positive charge but a small total positively charged surface area (high RPCG values), were ionized with better efficiency compared to MeOH. The partial charge had the greatest effect on the ionization (BCUTc-1h, RPCG, FPSA-3, DPSA-1). Both positive and negative partial charges were important, as a high difference between surface areas with partial positive and negative charge (DPSA-1) decreased ionization. The decreasing effect of relative negative charge surface area (RNCG) was attenuated by 1 mmol/L NH_3_. Compounds with high charge-weighted partial positive surface area and low total molecular surface area were ionized more efficiently (FPSA-3).

1 mmol/L NH_3_ as a makeup solvent in ESI^+^ should be preferred, as increasing the NH_3_ concentration decreased the MS responses for all compounds (Figure 3c). The molecular descriptor ANN- assigned weights at each concentration closely correlated with R^2^ > 0.6 and usually even >0.9 (SI 1 Table S3). The molecular descriptors with the highest SD were related to the molecular weight (BCUTw-1h) and distribution (Weta). In the next step, 1 mmol/L NH_3_ vs 1 mmol/L ammonium formate (AmF) vs 1 mmol/L ammonium acetate (AmAc) were compared. Molecular descriptor weights were highly correlated between NH_3_ and ammonia salts with R^2^ > 0.93 (SI 1 Table S3), confirmed by similar MS responses (+/– 20%) with some outliers. Only some molecular descriptors played a decisive role. In particular, the use of 1 mmol/L AmF instead of 1 mmol/L NH_3_ decreased the negative effect of BCUTw-1h, MOMI-Z, and MOMI-XY and increased the positive effect of nBase and Weta2. As a result, 1 mmol/L AmF should be preferred for SFC-MS analysis of basic, high molecular weighted spherical compounds.

The increasing NH_3_ concentration up to 5 mmol/L had negligible effect on the MS responses in APCI^+^ (within +/– 20% of the responses obtained using 1 mmol/L NH_3_). Higher NH_3_ concentrations predominantly increased the ionization. The negative effect of AlogP2 increased with increasing NH_3_ concentration. The positive effect of BCUTw-1h decreased at concentrations exceeding 5 mmol/L. The weights of charge-related descriptors DPSA-1 and WNSA-3 showed a U-profile behavior with the most pronounced effect at 5–10 mmol/L NH_3_. Again, spherical compounds were ionized to a lesser extent, with the negative effect becoming more pronounced with increasing concentration of NH_3_. 10 mmol/L AmF and AmAc instead of 10 mmol/L NH_3_ increased the ionization efficiency for over 95% of the compounds. This enhanced ionization was particularly significant in the case of AmF where as much as 60-fold higher responses were observed. The use of AmF mitigated the negative effect of sphericity on ionization (no effect of geomShape and positive weight of MOMI-Z) and, on the contrary, the positive effect of positive surface area (RPCS, PPSA-1) and secondary amines (khs.ssNH) became more pronounced.

### Ionization in SFC-MS Using 10 mmol/L Ammonia in Methanol as Organic Modifier in Positive Ionization Mode

Ammonia is commonly used additive to the SFC mobile phase to elute compounds with acid-basic properties in narrow symmetrical peaks. However, the presence of NH_3_ ions also affects the ionization in the MS source.

#### Methanol as Makeup Solvent

The comparison of key molecular descriptors selected by ANN shows that different molecular descriptors are important in the ionization process when MeOH and MeOH+NH_3_ are used as organic modifiers.

The charged state of the molecule played a role in the ionization processes, but lower charged surface areas were beneficial using MeOH+NH_3_ (BCUTc-1l). PNSA-3 with a decreasing effect in ESI^+^ using MeOH had an opposite effect using MeOH+NH_3_ (SI 1 Figure S5a). The same but more pronounced effect was observed for APCI^+^. The critical effect of the negatively charged surface area in APCI^+^ was further confirmed by the high weight assigned to the WNSA-3 (SI 1 Figure S5b). FPSA-3 increased the MS response using both ionization sources. However, the correlation between positively and negatively charged surface area and its effect on ionization changed with the change in the organic modifier for APCI^+^. Indeed, the positive effect of DPSA-1 remained the same in ESI^+^ using both organic modifiers, whereas it had a negligible effect in APCI^+^ using MeOH and a significant negative effect using MeOH+NH_3_. Similar trends were observed for WPSA-1. Moreover, a high relative positive charge surface area (RPCS) had a strong negative effect in ESI^+^. Many of the key molecular descriptors affecting the ionization were related to the distribution of atoms (SI 1 Figure S5a,b). Molecules with long chains (nAtomLC, nAtomLAC) and numerous rings (nRingBlocks), e.g., 6-atom rings (nRings6), had decreased MS responses in ESI^+^, while 7-atom rings had a positive effect with both ionization sources. The MS response in ESI^+^ decreased with the increasing number of primary amines (khs.sNH2). This was further confirmed by the more efficient ionization of compounds with large molecular distance edges between primary and secondary or tertiary amines (MDEN-12, MDEN-13). On the contrary, a higher number of secondary and tertiary amines improved the ionization, as shown by a positive effect of short molecular distance edges between them (negative weights of MDEN-23, MDEN-33). Aromatically bonded nitrogens (khs.aaNH) decreased ionization in APCI^+^ (SI 1 Figure S5b).

Secondary oxygens (khs.ssO, MDEO-22) decreased ionization in ESI^+^. The negative weight of MDEO-12 suggests a slightly positive effect of primary oxygens. Enhanced ionization was associated with aromatically bonded oxygens (khs.aaO). Aromatically bonded oxygens had a similar positive effect in APCI^+^. The MS response in APCI^+^ increased with chlorine substitution (khs.sCl), sulphone substitution (khs.ddssS), and =CH– groups (khs.dsCH). Quaternary carbons with double bonds (khs.aasC and khs.dssC) decreased the ionization efficiency. Molecular size (Wlambda1, BCUTw-1h,) affected the ionization in APCI^+^ to a greater extent (SI 1 Figure S5b). However, the increasing molecular weight must be related to the increasing number of polar groups, as the ionization in APCI^+^ decreased with increasing AlogP. The effect of polar groups in ESI^+^ is represented by the LipinskiFailure descriptor, where more efficient ionization is achieved for compounds that violate Lipinski′s rule. The ionization can be supported by a high number of basic groups (nBase), while a high number of acidic groups had a degrading effect (SI 1 Figure S3a). The NH_4_^+^ ions in the mobile phase also affected the preferences for the 3D shape of the molecule. Practically opposite behavior was observed for MOMI-Z and MOMI-XY when using MeOH and MeOH+NH_3_ as an organic modifier in both ion sources. Compounds with a spherical shape (geomShape) had decreased ionization efficiency using MeOH+NH_3_ in both ESI^+^ and APCI^+^, whereas compounds with 2D acyclic shape (topoShape) had increased ionization efficiency in APCI^+^.

#### The Effect of Alcohol Type

EtOH and IpOH as makeup solvents with MeOH+NH_3_ modifier provided comparable effects in ESI and APCI (SI 1 Figure S5c). IpOH was beneficial in ESI^+^ and APCI^+^, with about 70% of the compounds having higher MS responses, while mostly decreased MS responses were observed with EtOH. The standard deviations between the ANN weights (SI 1 Figure S3c) confirmed the smaller differences observed between the methanolic modifier and MeOH+NH_3_. For the modifier with NH_3_, the negative effect of RPCG and RNCG in ESI^+^ increased with EtOH and IpOH (SI 1 Figure S5a). The partial charge and surface area related molecular descriptors were mainly affected in APCI^+^ (PNSA-3, WNSA-3), and the effect of aromatically bonded oxygens (khs.aaO) was reversed (SI 1 Figure S5b). IpOH was beneficial for ionization in APCI^+^ for highly lipophilic compounds (ALogP2) with high RNCS.

#### The Effect of Additive

##### Water

1 mmol/L H_2_O had mostly positive effect in ESI^+^ whereas the effect was strongly dependent on the analyte properties in APCI^+^ (SI 1 Figure S6a). The violin plots also showed low SD between molecular descriptor weights for MeOH vs 1 mmol/L H_2_O in ESI^+^ and significant differences in APCI^+^ (SI 1 Figure S4c).

1 mmol/L H_2_O increased the positive effect of the negative surface area of the molecule in ESI^+^ (WNSA-3 and RNCS, SI 1 Figure S5a). Overall, similar ionization processes occurred with R^2^ of 0.8 between the molecular descriptor rankings for MeOH and 1 mmol/L H_2_O (SI 1 Figure S6b). The effect of negatively charged surface area (PNSA-3, WNSA-3) decreased in APCI^+^ with H_2_O in the makeup solvent. The RNCS became the most important molecular descriptor and significantly decreased ionization, while the RPCS, ranked as the third descriptor, increased ionization (SI 1 Figure S5b), which completely changed the ionization efficiency compared to the methanolic makeup solvent (SI 1 Figure S6c).

Higher concentrations of H_2_O increased MS responses for approx. 50% of compounds in ESI^+^. Nevertheless, concentrations over 1% resulted in a strong decrease for several compounds (SI 1 Figure S6d). Similar weights were assigned to the molecular descriptors using all tested H_2_O concentrations except for 5%. In APCI^+^, 5 mmol/L H_2_O had a contradictory effect based on the analyte properties, with similar trends even at higher H_2_O concentrations (SI 1 Figure S6e). The H_2_O concentration effect on descriptors related to lipophilicity and molecular weight was reduced.

#### Acids

The use of 1 mmol/L FA was detrimental for most MS responses in ESI^+^, whereas similar responses occurred in APCI^+^ (SI 1 Figure S6a). ANN-assigned weights of molecular descriptors remained similar to those obtained using MeOH in ESI^+^ with higher SD in APCI^+^ (SI 1 Figure S6b,c). Changing the makeup solvent from MeOH to 1 mmol/L FA had less effect on the ionization when using MeOH+NH_3_ as organic modifier instead of MeOH for both sources (R^2^ 0.4). In ESI^+^, the positive effect of molecular weight (BCUTw-1h), secondary and aromatic oxygens (khs.aaO, MDEO-22), and basic groups was attenuated (SI 1 Figure S5a), while low polarizability (BCUTp-1l), carbon connectivity (khs.aasC), and molecular distribution (Weta3.u) became more pronounced. However, mostly the same molecular descriptors are among the key ones that positively affect the ionization using both MeOH and 1 mM FA, including positive surface area (DPSA-1, WPSA-1), sphericity (MOMI-Z, geomShape), and non-compliance with Lipinski rules (SI 1 Figure S5a). In APCI^+^, 1 mmol/L FA attenuated the positive effect of low negative charge (WNSA-3, PNSA-3) and the negative effect of positive charge (DPSA-1). A high relative negative charge (RNCG) was beneficial for ionization (SI 1 Figure S5b), corresponding to a beneficial effect of a lower number of acidic groups (nAcid). Strongly lipophilic compounds ionized more efficiently (AlogP2) (SI 1 Figure S5b).

The use of 5 mmol/L FA instead of 1 mM is recommended in ESI^+^ with an ammonium-based modifier, as responses increased for most compounds. Further increases in FA concentration had a negligible effect (SI 1 Figure S6d). The increased FA concentration enhanced the positive effect of low negative charge (PNSA-3, WNSA-3), basic groups (nBase), and molecular weight (BCUTw-1h) and mitigated the negative effect of acidic groups (nAcid). Switching to AA was beneficial for basic compounds (nBase) with large surface areas with a partial positive charge (RPCS). Different concentrations of FA behaved similarly in APCI, except for 10 mmol/L, 20 mmol/L, and 0.1%, where different molecular descriptors became more important (SI 1 Figure S5b), requiring careful optimization of FA concentration when using APCI^+^. Again, mostly U-shaped trends were observed.

#### Ammonia

Only small changes in ionization were expected by changing MeOH to 1 mmol/L NH_3_ in MeOH as a makeup solvent when MeOH+NH_3_ was used as an organic modifier. This was true for APCI^+^ with mostly similar MS responses (SI 1 Figure S6a). However, the further addition of NH_3_ was beneficial for ESI^+^ (SI 1 Figure S6a). These results correspond with violin plots of SD between molecular descriptor weights for MeOH and 1 mmol/L NH_3_. For APCI, all SD were <1.5, with slightly higher values for ESI (SI 1 Figure S4).

The ammonia ions and alkoxyl carbamates from the mobile phase can interact with amine and oxygen groups, which explains the mitigation of the negative effect of these groups (khs.sNH2, khs.ssO) in ESI^+^. Molecules containing cyclic moieties (high FMF values) were ionized more efficiently. Otherwise, the ionization remained similar with the presence of basic functional groups and a high total positive charge surface increasing ionization. The change to 5 mmol/L NH_3_ had almost no effect in ESI^+^, but higher concentrations of NH_3_ in makeup solvent mainly decreased the ionization efficiency. 1 mmol/L NH_3_ is again recommended for efficient ionization of most compounds in ESI^+^. Increasing NH_3_ concentration primarily changed the weights of molecular descriptors related to lipophilicity (ALogP2), molecular weight (BCUTw-1h), negatively charged surface (FNSA-3), and 3D shape (MOMI-XY, MOMI-Z, geomShape). A comparison of 1 mmol/L NH_3_ vs 1 mmol/L AmF and 1 mmol/L AmAc showed that the use of AmF resulted in decreased MS responses for over 90% of the compounds, but only for 50% of the compounds using AmAc and usually within +/– 20%.

Negligible changes in ionization were observed in APCI^+^ with nearly the same ranking of most molecular descriptors (SI 1 Figure S6c). Only a slightly higher positive effect of the RNCS was observed (SI 1 Figure S5b). Increasing NH_3_ concentration resulted in similar and/or higher MS responses. However, this increase was prevalent for most compounds only when using 100 mmol/L NH_3_ in MeOH (SI 1 Figure S6e), which should be the concentration used at the beginning of method development in APCI^+^. The weights of most of the key molecular descriptors changed gradually with increasing concentration of NH_3_, i.e., AlogP, BCUTw-1h, PNSA-3, WNSA-3, MOMI-Z, MOMI-XY (SI 1 Figure S5b). In contrast to ESI^+^, the use of ammonium salts was beneficial in APCI^+^. The use of 10 mM AmF and 10 mM AmAc had both decreasing and increasing effects on MS responses. However, the MS responses were mostly decreased only by up to 20%, whereas increases of up to 60-fold were observed.

The reader is referred to SI 2 for the discussion and results of ionization in negative mode.

## Conclusion

The application of ANN provides exceptional capability for identifying correlations within large datasets, based on strict rules established during training cycles. In this study, we utilized ANN to gain insights into the ionization processes occurring in ESI and APCI under varying makeup solvent conditions. In ESI^+^, analytes with high positive partial surface charge, small negatively charged areas, low molecular weight, and spherical shapes ionized most effectively. Linear structures showed lower responses in both ESI^+^ and APCI^+^, while APCI^+^ favored asymmetric molecules with concentrated positive surface areas and secondary or tertiary amines. Ionization in APCI^−^ and ESI^−^ showed similar behavior, with improved ionization when surface charge, especially positive, was localized. Linear and elongated molecules were more efficiently ionized in negative mode. With MeOH+NH_3_ as organic modifier, descriptors linked to nitrogen and oxygen bonding gained importance, especially in ESI^+^, where abundant NH_4_^+^ facilitates efficient protonation at basic sites. Both positively and negatively charged surfaces contributed to ionization, with positive charge being more favorable in ESI^+^ and localized negative charge aiding APCI^+^. APCI^+^ ionization also correlated with molecular size but not lipophilicity, underlining the role of polar functional groups. In ESI^−^, features like partial negative charge, hydrogen bond donors, and primary oxygens supported ionization. In APCI^−^, positively charged surfaces were more beneficial, likely due to their role in intermolecular interactions during ion formation. Our results strongly encourage the use of other alcohols as makeup solvents in positive ionization mode, regardless of the organic modifier used. MeOH should be preferred in ESI^−^, while improved ionization efficiency in APCI^−^ was observed for EtOH and IpOH. The addition of ammonia to the modifier slightly attenuated the changes in the ionization efficiency, but the use of IpOH instead of MeOH can be recommended for both ESI^+^ and APCI^+^. Overall, the use of an additive alters the effects of the partially charged surface area, i.e., a site available for interactions with the molecules of the additive, affecting the transfer of protons and also the transfer to the gas phase by hydration of the analyte. The use of an additive also changes the apparent pH of the eluent entering MS as shown by the 1 mmol/L FA which increased the importance of the partial positive charge for ionization in APCI^+^, which may be related to the more pronounced acidic properties of the solvent entering the MS, allowing easier transfer of the H^+^.

These findings deepen the understanding of the factors affecting ionization efficiency in SFC-MS workflows and can serve as a valuable proof-of-concept for the development of predictive models. Indeed, these results will be validated in future studies to enable the creation of prediction models that enable to select the optimal makeup solvent composition. Nevertheless, such prediction modeling needs further investigation focusing on additional parameters. This fundamental understanding enables data-driven solvent selection, eliminating trial-and-error approaches and accelerating method development, especially for complex matrices or novel compounds. Ultimately, this approach has the potential to revolutionize method optimization in SFC-MS, enabling more precise and efficient *in silico* predictions of analyte behavior under various analytical conditions, and paving the way for broader applications of SFC-MS methods in analytical chemistry.

## Supplementary Material





## Data Availability

Data availability statement The original data used in this publication are openly available at Zenodo under the doi: 10.5281/zenodo.15355651.

## Notes

**SI 1.** List of analytes; List of molecular descriptors; Equations-calculation of measured-adjusted responses; Description of the moment of inertia; Violin plots of standard deviations; Correlation coefficients. (PDF)
**SI 2.** Results and discussion for negative ionization mode. (PDF)

(PDF)
(PDF)
