# Supplementary material for: Artificial Neural Networks: An Innovative Approach Used for Elucidation of Ionization Processes in Supercritical Fluid Chromatography-Mass Spectrometry
Source: Anal Chem. 2025 May 10;97(19):10252–63. doi: 10.1021/acs.analchem.5c00152 (PMC12096348; doi:10.1021/acs.analchem.5c00152)
Supplement: Supplementary file 1 [file ac5c00152_si_001.pdf]

Supporting Information 1: Artificial neural networks: an innovative approach used for elucidation of ionization processes in supercritical fluid chromatography-mass spectrometry

Kateřina Plachká[1], Veronika Pilařová[1], Taťána Gazárková[1], Jean-Christophe Garrigues[2], František Švec[1], Lucie Nováková[1*]

[1] Department of Analytical Chemistry, Faculty of Pharmacy in Hradec Králové, Charles University, Akademika Heyrovského 1203/8, 50003 Hradec Králové, Czechia

[2] SOFTMAT (IMRCP) Laboratory, SMODD Team, CNRS, Toulouse III Paul Sabatier University, 31400 Toulouse, France

* Email: nol@email.cz (Lucie Nováková)

Table of Content

Methods

Table S1. List of analytes detected at specific SFC-MS conditions.

Table S2. Molecular descriptors used in the study, calculated with CDK Descriptor Calculator (v.1.4.8).

Chromatographic conditions.

Equations S1. Equations used for the calculation of measured-adjusted responses adjusted from Plachka et al.[1]

Results

Figure S1. Description and general representation of the moment of inertia (MOMI) molecular descriptor.

Figure S2. The effect of ethanol (EtOH) and isopropanol (IpOH) as makeup solvents on MS responses compared to MS responses obtained with MeOH.

Figure S3. Violin plots summarizing standard deviations (SD) between weights assigned by ANN to the molecular descriptors when using ethanol (EtOH) and propan-2-ol (IpOH) instead of methanol (MeOH)

as makeup solvent. Conditions: a – MeOH as organic solvent, positive ionization mode, b – MeOH as organic modifier, negative ionization mode, c – 10 mmol/L NH_3 in MeOH, positive ionization mode, d – 10 mmol/L NH_3 in MeOH, negative positive mode.

Figure S4. Violin plots summarizing standard deviations (SD) between weights assigned by ANN to the molecular descriptors when using 1mM H_2O in methanol, 1mM NH_3 in methanol, and 1mM formic acid (FA) instead of methanol (MeOH) as makeup solvent. Conditions: a – MeOH as organic solvent, positive ionization mode, b – MeOH as organic modifier, negative ionization mode, c – 10 mmol/L NH_3 in MeOH, positive ionization mode, d – 10 mmol/L NH_3 in MeOH, negative positive mode.

Figure S5. Key molecular descriptors affecting ionization in ESI^+ (a) and $APCI^+$ (b) when using 10 mmol/L NH_3 in MeOH as organic modifier. The shade of blue in the heatmaps corresponds to the ranking of the molecular descriptor for each makeup solvent composition (ranking 1 = the highest absolute ANN-assigned weight = the darkest blue). ↑- increasing effect on ionization, ↓-decreasing effect on ionization. (c) The effect of ethanol (EtOH) and isopropanol (IpOH) as makeup solvents on MS responses compared to MS responses obtained using MeOH.

Figure S6. Effect of 1 mmol/L additive in makeup solvent compared to pure methanol as makeup solvent on MS responses (a) and a comparison of rankings of molecular descriptors based on ANN-assigned weights (b, c) for ESI^+ (blue) and $APCI^+$ (orange). Effect of increasing additive concentration on MS responses in ESI^+ (d) and $APCI^+$ (e) compared to MS responses obtained using 1 mmol/L additive in methanol. Over 10-fold lower (dark red), 10-5-fold lower (red), 5-2-fold lower (dark pink), 0.2-1-fold lower (pink), within +/- 20% (orange), 0.2-1-fold higher (the lightest blue), 2-5-fold higher (light blue), 5-10-fold higher (blue), over 10-fold higher (dark blue). All results were obtained using 10 mmol/L ammonia in methanol as the organic modifier.

Table S3. Correlation coefficients between molecular descriptor weights assigned by ANN based on makeup solvents with various concentrations of additives compared to 1 mM.

Table S1: List of analytes detected at specific SFC-MS conditions. ACN – acetonitrinile; MeOH – methanol, N – not detected, THF – tetrahydrofuran, y – detected.

				Organic modifier: MeOH																				Organic modifier: MeOH+NH_3																			
				ESI^+					ESI^-					$APCI^+$					$APCI^-$					ESI^+					ESI^-					$APCI^+$					$APCI^-$				
n.	CAS No.	analyte	solvent	alcohols	ammonia	buffer	acidic	water	alcohols	ammonia	buffer	acidic	water	alcohols	ammonia	buffer	acidic	water	alcohols	ammonia	buffer	acidic	water	alcohols	ammonia	buffer	acidic	water	alcohols	ammonia	buffer	acidic	water	alcohols	ammonia	buffer	acidic	water	alcohols	ammonia	buffer	acidic	water
2	2614-06-4	(+)-thalidomide	ACN	n	N	n	n	n	n	n	n	n	n	y	y	y	y	y	y	y	y	y	y	n	n	n	n	n	n	n	n	n	n	y	y	y	y	y	y	y	y	y	y
3	303-38-8	2,3-dihydroxy benzoic acid	ACN	n	N	n	n	n	n	n	n	n	n	n	n	n	n	n	n	n	n	n	n	n	n	n	n	n	n	y	n	y	y	n	n	n	n	n	n	n	n	n	n
4	89-86-1	2,4-dihydroxy benzoic acid	ACN	n	N	n	n	n	n	n	n	n	n	n	n	n	n	n	n	n	n	n	n	n	n	n	n	n	n	y	n	y	y	n	n	n	n	n	n	n	n	n	n
5	490-79-9	2,5-dihydroxy benzoic acid	ACN	n	N	n	n	n	n	n	n	n	n	n	n	n	n	n	n	n	n	n	n	n	n	n	n	n	n	y	y	y	y	n	n	n	n	n	n	n	n	n	n
6	303-07-1	2,6-dihydroxy benzoic acid	ACN	n	N	n	n	n	n	n	n	n	n	n	n	n	n	n	n	n	n	n	n	n	n	n	n	n	n	y	n	y	y	n	n	n	n	n	n	n	n	n	n
7	583-17-5	2-hydroxy cinnamic acid	ACN	n	Y	y	n	y	y	y	y	y	y	n	y	y	y	y	y	y	y	y	y	y	y	n	n	y	y	y	y	y	y	n	y	y	y	y	y	y	y	y	y
8	362-07-2	2-methoxyestradiol	ACN	y	Y	y	y	y	n	n	n	n	n	y	y	y	y	y	n	n	n	n	n	y	y	y	y	y	y	y	y	y	y	y	y	y	y	y	n	n	n	n	n
9	99-50-3	3,4-dihydroxy benzoic acid	ACN	n	N	n	n	n	n	n	n	n	n	n	n	n	n	n	n	n	n	n	n	n	n	n	n	n	n	y	n	y	y	n	n	n	n	n	n	n	n	n	n
10	99-10-5	3,5-dihydroxy benzoic acid	ACN	n	N	n	n	n	n	n	n	n	n	n	n	n	n	n	n	n	n	n	n	n	n	n	n	n	y	y	y	y	y	n	n	n	n	n	y	y	y	y	y
11	530-59-6	4-hydroxy-3,5-dimethoxy-cinnamic acid	ACN	n	Y	y	y	y	y	y	y	y	y	n	n	n	n	n	n	n	n	n	n	y	y	y	y	y	y	y	y	y	y	y	y	y	y	y	y	y	y	y	y
12	588-30-7	3-hydroxy cinnamic acid	ACN	n	N	n	n	n	y	y	y	y	y	n	n	n	n	n	y	y	y	y	y	n	n	n	n	n	y	y	y	y	y	n	n	n	n	n	y	y	y	y	y
13	537-73-5	3-hydroxy-4-methoxycinnamic acid	ACN	n	N	n	n	n	y	y	y	y	n	n	n	n	n	n	y	y	y	y	y	n	y	y	y	n	y	y	y	y	y	n	y	y	y	y	y	y	y	y	y
14	530-57-4	4-hydroxy-3,5-dimethoxybenzoic acid	ACN	n	N	n	n	n	n	n	n	n	n	n	n	n	n	n	n	n	n	n	n	y	y	n	y	y	y	y	y	y	y	n	n	n	n	n	n	n	n	n	n
15	121-34-6	4-hydroxy-3-methoxy benzoic acid	ACN	n	N	n	n	n	n	n	n	n	n	n	n	n	n	n	n	n	n	n	n	n	y	n	n	n	n	y	y	y	y	n	y	y	y	y	n	n	n	n	n
16	99-96-7	4-hydroxybenzoic acid	ACN	n	N	n	n	n	n	y	y	n	n	n	n	n	n	n	n	n	n	n	n	n	n	n	n	n	y	y	y	y	y	n	n	n	n	n	n	n	n	n	n
17	100-09-4	4-methoxy benzoic acid	ACN	n	N	n	n	n	n	y	y	n	n	n	n	n	n	n	n	n	n	n	n	n	n	n	n	n	y	y	y	y	y	n	n	n	n	n	y	y	y	y	y
18	154229-18-2	abiraterone acetate	ACN	y	Y	y	y	y	n	n	n	n	n	y	y	y	y	y	n	n	n	n	n	y	y	y	y	y	n	n	n	n	n	y	y	y	y	y	n	n	n	n	n
19	37517-30-9	acebutolol	MeOH/ACN	y	Y	y	y	y	y	y	y	y	y	y	y	y	y	y	n	y	n	n	y	y	y	y	y	y	y	y	y	y	y	y	y	y	y	y	y	y	y	y	y
20	138112-76-2	agomelatine	ACN	y	Y	y	y	y	y	y	y	y	y	y	y	y	y	y	y	y	y	y	y	y	y	y	n	y	y	y	y	y	y	y	y	y	y	y	n	n	y	y	y
21	52-39-1	aldosterone	ACN	n	N	n	n	n	n	n	n	n	n	n	n	n	n	n	y	y	y	y	y	n	n	n	n	n	n	n	n	n	n	y	y	y	y	y	y	y	y	y	y
22	59-02-9	alpha-tocopherol	ACN	y	Y	y	y	y	y	y	y	y	y	y	y	y	y	y	y	y	y	y	y	y	y	y	y	y	y	y	y	y	y	y	y	y	y	y	n	y	y	y	y
23	1721-51-3	alpha-tocotrienol	ACN	y	Y	y	y	y	n	y	y	y	n	y	y	y	y	y	y	y	y	y	y	y	y	y	y	y	y	y	n	y	y	y	y	y	y	y	y	y	y	y	y
24	520-36-5	apigenin	MeOH/ACN	n	N	n	n	n	n	n	n	n	n	n	n	n	n	n	n	n	n	n	n	n	n	n	n	n	y	y	y	y	y	n	n	n	n	n	y	y	y	y	y
25	29122-68-7	atenolol	MeOH/ACN	n	N	n	n	n	n	n	n	n	n	n	n	n	n	n	n	n	n	n	n	y	y	y	y	y	n	n	n	n	n	y	y	y	y	y	n	n	n	n	n
26	98-55-5	a-terpineol	ACN	n	N	n	n	n	n	n	n	n	n	n	n	n	n	n	n	n	n	n	n	n	n	n	n	n	n	n	n	n	n	n	n	n	n	n	n	n	n	n	n
27	83015-26-3	atomoxetine	MeOH/ACN	y	Y	y	y	y	n	n	n	n	n	n	y	n	n	y	n	n	n	n	n	y	y	y	n	y	n	n	n	n	n	y	y	y	y	y	n	n	n	n	n
28	134523-00-5	atorvastatin	MeOH	y	Y	y	y	y	y	y	y	y	y	y	y	y	y	y	y	y	y	y	y	y	y	y	y	y	y	y	y	y	y	y	y	y	y	y	y	y	y	y	y
29	148-03-8	beta-tocopherol	ACN	n	Y	y	y	y	y	y	y	y	y	y	y	y	y	y	y	y	y	y	y	y	y	y	y	n	y	y	y	y	y	y	y	y	y	y	y	y	y	y	y
30	490-23-3	beta-tocotrienol	ACN	y	Y	y	y	y	y	y	y	y	y	y	y	y	y	y	n	y	y	y	y	y	y	y	y	y	y	y	y	y	y	y	y	y	y	y	n	y	y	y	y
31	378-44-9	betamethasone	ACN	n	N	n	n	n	n	y	y	y	y	n	n	n	n	n	y	y	y	y	y	y	y	y	y	y	y	y	y	y	y	n	y	y	y	y	y	y	y	y	y
32	62658-63-3	bopindolol	ACN	y	Y	y	y	y	y	y	y	y	y	n	y	n	n	y	n	y	n	n	y	y	y	y	n	y	y	y	y	y	y	y	y	y	y	y	y	y	y	y	y

33	51-20-7	bromouracil	MeOH/ACN	n	N	n	n	n	y	y	y	y	y	n	n	n	n	n	y	y	y	y	y	n	n	n	n	n	y	y	y	y	y	n	n	n	n	n	y	y	y	y	y
34	58-08-2	caffeine	MeOH/ACN	y	Y	y	y	y	n	n	n	n	n	y	y	y	y	y	n	n	n	n	n	y	y	y	n	y	n	n	n	n	n	y	y	y	y	y	n	n	n	n	n
36	83881-51-0	cetirizine	MeOH/ACN	n	Y	n	n	y	n	n	n	n	n	n	n	n	n	n	n	n	n	n	n	y	y	y	y	y	y	y	y	y	y	y	y	y	y	y	y	y	y	y	y
37	50-22-6	corticosterone	ACN	n	N	n	n	n	n	n	n	n	n	y	y	y	y	y	y	y	y	y	y	n	n	n	n	n	n	n	n	n	n	y	y	y	y	y	y	y	y	y	y
39	1009119-64-5	daclatasvir	ACN	n	Y	y	y	y	n	n	n	n	n	n	y	y	y	n	n	n	n	n	n	y	y	y	y	y	n	y	y	y	y	y	y	y	y	y	n	y	y	y	y
40	133099-04-4	darifenacin	MeOH/ACN	y	Y	y	y	y	n	n	n	n	n	n	y	n	n	n	n	n	n	n	n	y	y	y	n	y	y	n	n	n	n	y	y	y	y	y	y	y	n	y	y
41	302962-49-8	dasatinib	MeOH/ACN	n	N	n	n	n	n	n	n	n	n	n	n	n	n	n	n	n	n	n	n	y	y	y	y	y	n	y	y	y	y	y	y	y	y	y	n	n	n	n	n
42	53-43-0	dehydro-epi-androsterone	ACN	n	N	n	n	n	n	n	n	n	n	n	n	n	n	n	n	n	n	n	n	n	n	n	n	n	n	n	n	n	n	y	y	y	n	y	n	n	n	n	n
43	119-13-1	delta-tocopherol	ACN	y	Y	y	y	y	n	y	y	y	y	y	y	y	y	y	y	y	y	y	y	y	y	y	y	y	y	y	y	y	y	y	y	y	y	y	n	y	y	y	y
44	25612-59-3	delta-tocotrienol	ACN	n	Y	y	y	y	y	y	y	y	y	y	y	y	y	y	y	y	y	y	y	y	y	y	y	y	y	y	y	y	y	y	y	y	y	y	y	y	n	y	y
45	50-47-5	desipramine	MeOH/ACN	n	N	n	n	n	n	n	n	n	n	n	n	n	n	n	n	n	n	n	n	y	y	y	n	y	n	n	n	n	n	y	y	y	y	y	n	n	n	n	n
46	50-02-2	dexamethasone	ACN	y	Y	y	y	y	n	n	n	n	n	n	y	y	y	n	y	y	y	y	y	y	y	y	y	y	y	y	y	y	y	y	y	y	y	y	y	y	y	y	y
47	915087-33-1	enzalutamide	ACN	y	Y	y	y	y	y	y	y	y	y	y	y	y	y	y	y	y	y	y	y	y	y	y	y	y	y	y	y	y	y	y	y	y	y	y	y	y	y	y	y
48	57-91-0	estradiol	ACN	n	N	n	n	n	y	y	n	y	y	n	n	n	n	n	y	y	y	y	y	n	n	n	n	n	y	y	y	y	y	n	n	n	n	n	y	y	n	y	y
49	4245-41-4	estradiol acetate	ACN	y	Y	y	y	y	n	n	y	n	n	y	y	y	y	y	n	n	n	n	n	y	y	y	y	y	n	n	n	n	n	y	y	y	y	y	n	y	y	y	y
50	50-27-1	estriol	MeOH/ACN	y	Y	y	y	y	y	y	y	y	y	n	y	y	y	y	y	y	y	y	y	y	y	y	y	y	y	y	y	y	y	y	y	y	y	y	y	y	y	y	y
51	53-16-7	estron	MeOH/ACN	y	Y	y	y	y	y	y	y	y	y	y	y	y	y	y	y	y	y	y	y	y	y	y	y	y	y	y	y	y	y	y	y	y	y	y	y	y	y	y	y
52	774-40-3	(±)-ethyl mandelate	ACN	n	N	n	n	n	n	n	n	n	n	n	n	n	n	n	n	n	n	n	n	y	y	y	y	y	n	y	y	y	n	n	y	y	y	y	n	n	n	n	n
54	163222-33-1	ezetimibe	ACN	y	Y	y	y	y	y	y	y	y	y	y	y	n	y	y	y	y	y	y	y	n	y	y	y	y	y	y	y	y	y	n	y	y	y	y	y	y	y	y	y
55	4602-84-0	farnesol	ACN	y	Y	y	y	y	n	y	n	n	n	y	y	y	n	y	n	n	n	n	n	n	n	n	n	n	n	y	n	n	n	n	y	y	y	y	n	n	n	n	n
56	29679-58-1	fenoprofen	MeOH/ACN	n	N	n	n	n	n	y	n	n	n	n	n	n	n	n	n	y	n	n	y	n	n	n	n	n	y	y	y	y	y	n	n	n	n	n	y	y	y	y	y
57	5104-49-4	flurbiprofen	ACN	n	N	n	n	n	n	n	n	n	n	n	n	n	n	n	n	n	n	n	n	n	n	n	n	n	n	y	y	y	y	n	y	y	y	y	y	y	y	y	y
58	93957-54-1	fluvastatin	MeOH/ACN	y	Y	y	y	y	y	n	n	n	n	n	y	y	y	y	n	n	n	n	n	y	y	y	y	y	y	y	y	y	y	y	y	y	y	y	n	y	y	y	y
59	54-28-4	gamma-tocopherol	ACN	y	Y	y	y	y	n	y	y	y	y	y	y	y	y	y	n	y	y	y	y	y	y	y	y	y	y	y	y	y	y	y	y	y	y	y	y	y	y	y	y
60	14101-61-2	gamma-tocotrienol	ACN	y	Y	y	y	y	n	y	y	y	y	y	y	y	y	y	y	y	y	y	y	y	y	y	y	y	y	y	y	y	y	y	y	y	y	y	n	y	y	y	y
61	520-33-2	hesperetin	MeOH/ACN	n	Y	y	y	n	n	n	n	n	n	y	y	y	y	y	y	y	y	y	y	y	y	y	y	y	y	y	y	y	y	y	y	y	y	y	y	y	y	y	y
62	520-26-3	hesperidin	MeOH/ACN	n	N	n	n	n	n	n	n	n	n	n	n	n	n	n	n	n	n	n	n	y	y	n	n	y	y	y	y	y	y	n	y	y	y	y	n	y	y	y	y
63	4270-27-3	chlorouracil	MeOH/ACN	n	N	n	n	n	y	y	y	y	y	n	n	n	n	n	y	y	y	y	y	n	n	n	n	n	y	y	y	y	y	n	n	n	n	n	y	y	y	y	y
64	15687-27-1	ibuprofen	ACN	n	N	n	n	n	n	n	n	n	n	n	n	n	n	n	n	n	n	n	n	y	y	y	n	y	n	y	y	y	y	n	y	y	y	y	n	y	y	y	y
65	50-49-7	imipramine	MeOH/ACN	y	Y	n	n	n	n	n	n	n	n	n	y	n	n	y	n	n	n	n	n	y	y	y	y	y	n	n	n	n	n	y	y	y	y	y	n	n	n	n	n
66	1516864-05-3	atorvastatin impurity A	MeOH/ACN	y	Y	y	y	y	y	y	y	y	y	y	y	y	y	y	n	y	y	y	y	y	y	y	y	y	y	y	y	y	y	y	y	y	y	y	y	y	y	y	y
67	842103-12-2	atorvastatin impurity B	MeOH/ACN	y	Y	y	y	y	y	y	y	y	y	y	y	n	y	y	y	y	y	y	y	y	y	y	y	y	y	y	y	y	y	y	y	y	y	y	y	y	y	y	y
68	693793-53-2	atorvastatin impurity C	MeOH/ACN	y	Y	y	y	y	y	y	y	y	y	y	y	y	y	y	y	y	y	y	y	y	y	y	y	y	y	y	y	y	y	y	y	y	y	y	y	y	y	y	y
69	53-86-1	indomethacin	ACN	y	Y	y	y	y	y	y	y	y	y	y	y	y	y	y	n	y	y	y	y	y	y	y	y	n	y	y	y	y	y	n	y	y	y	y	n	y	y	y	y
71	22071-15-4	ketoprofen	ACN	n	Y	y	y	y	n	y	n	n	n	y	y	y	y	y	n	y	y	y	y	y	y	y	y	n	y	y	y	y	y	y	y	y	y	y	n	y	y	y	y
72	36894-69-6	labetalol	MeOH/ACN	n	N	n	n	n	n	n	n	n	n	n	n	n	n	n	n	n	n	n	n	y	y	y	y	y	y	y	n	y	y	y	y	y	y	y	y	y	y	y	y
73	125995-03-1	atorvastatin lactone	ACN	y	Y	y	y	y	n	n	n	n	n	y	y	y	y	y	y	y	y	y	y	y	y	y	y	y	y	y	y	y	y	y	y	y	y	y	n	y	y	y	y
74	1256388-51-8	ledipasvir	MeOH/ACN	y	Y	y	y	y	n	n	n	n	n	y	y	y	y	y	y	y	y	y	y	y	y	y	y	y	n	n	n	n	n	y	y	y	y	y	y	y	y	y	y
75	75330-75-5	lovastatin	ACN	y	Y	y	y	y	n	y	y	y	n	y	y	y	y	y	y	y	y	y	y	y	y	y	y	y	y	y	y	y	y	y	y	y	y	y	y	y	y	y	y
76	491-70-3	luteolin	MeOH/ACN	n	N	n	n	n	n	n	n	n	n	n	n	n	n	n	n	n	n	n	n	n	n	n	n	n	n	n	n	n	n	n	n	n	n	n	n	n	n	n	n
77	376348-65-1	maraviroc	ACN	n	N	n	n	n	n	y	y	y	n	n	n	n	n	n	n	y	y	y	y	y	y	y	y	y	y	y	y	y	y	y	y	y	y	y	y	y	y	y	y
78	51384-51-1	metoprolol	MeOH/ACN	n	N	n	n	n	n	n	n	n	n	n	n	n	n	n	n	n	n	n	n	y	y	y	n	n	y	y	y	y	y	y	y	y	y	y	n	n	n	n	n
83	13523-86-9	pindolol	MeOH/ACN	n	N	n	n	n	n	y	y	y	n	n	n	n	n	n	n	y	n	y	y	y	y	y	y	y	y	y	y	y	y	y	y	y	y	y	y	y	y	y	y

84	147511-69-1	pitavastatin	MeOH	n	N	n	n	n	n	n	n	n	n	y	y	y	y	y	y	y	y	y	y	n	n	n	n	n	n	n	n	n	n	y	y	y	y	y	y	y	y	y	y
85	81093-37-0	pravastatin	MeOH	n	N	n	n	n	n	n	n	n	n	n	n	n	n	n	y	y	y	y	y	n	n	n	n	n	n	n	n	n	n	n	n	n	n	n	y	y	y	y	y
86	525-66-6	propranolol	MeOH/ACN	n	N	n	n	n	n	n	n	n	n	n	n	n	n	n	n	n	n	n	n	y	y	y	n	y	n	n	n	n	n	y	y	y	y	y	n	n	n	n	n
87	501-36-0	resveratrol	MeOH/ACN	n	N	n	n	n	y	y	y	y	y	n	n	n	n	n	y	y	y	y	y	n	y	n	y	n	y	y	y	y	y	y	y	y	y	y	y	y	y	y	y
88	155213-67-5	ritonavir	MeOH/ACN	y	Y	y	y	y	y	y	y	y	n	y	y	y	y	y	n	y	y	y	y	y	y	y	y	y	y	y	y	y	y	y	y	y	y	y	n	y	y	y	y
92	92-61-5	scopoletin	MeOH/ACN	y	Y	y	y	y	y	y	y	y	y	y	y	y	y	y	y	y	y	y	y	y	y	y	n	y	y	y	y	y	y	y	y	y	y	y	y	y	y	y	y
93	138-59-0	shikimic acid	MeOH/ACN	n	N	n	n	n	n	n	n	n	n	n	n	n	n	n	n	n	n	n	n	n	n	n	n	n	n	y	y	y	y	n	n	n	n	n	y	y	y	y	y
94	923604-59-5	simeprevir	MeOH/ACN	n	N	n	n	n	y	y	y	y	y	n	n	n	n	n	y	y	y	y	y	y	y	y	y	y	y	y	y	y	y	y	y	y	y	y	y	y	y	y	y
95	79902-63-9	simvastatin	ACN	n	N	n	n	n	y	y	y	y	y	n	y	n	y	y	y	y	y	y	y	y	y	y	y	y	y	y	n	y	y	y	y	y	y	y	n	y	y	y	y
96	486460-32-6	sitagliptin	MeOH/ACN	y	Y	y	n	y	n	y	y	y	y	n	n	n	n	n	n	n	n	n	n	y	y	y	y	y	y	y	y	y	y	y	y	y	y	y	y	y	y	y	y
97	1190307-88-0	sofosbuvir	MeOH/ACN	y	Y	y	y	y	y	y	y	y	y	y	y	y	y	y	y	y	y	y	y	y	y	y	y	y	y	y	y	y	y	y	y	y	y	y	y	y	y	y	y
99	611-40-5	tectoridin	MeOH	n	N	n	n	n	n	n	n	n	n	n	n	n	n	n	n	n	n	n	n	y	y	y	y	y	n	y	y	y	y	n	y	y	y	y	y	y	y	y	y
101	58-22-0	testosterone	ACN	n	N	n	n	n	n	n	n	n	n	y	y	y	y	y	n	y	n	n	y	n	n	n	n	n	n	n	n	n	n	y	y	y	y	y	n	y	y	n	y
102	274693-27-5	ticagrelor	ACN	n	N	n	n	n	n	n	n	n	n	y	y	y	y	y	y	y	y	y	y	n	n	n	n	n	n	n	n	n	n	y	y	y	y	y	y	y	y	y	y
103	140-10-3	trans-cinnamic acid	MeOH/ACN	n	N	n	n	n	n	n	n	n	n	n	n	n	n	n	n	y	y	y	y	n	n	n	n	n	y	y	n	y	n	n	y	y	y	y	y	y	y	y	y
104	501-94-0	tyrosol	ACN	n	N	n	n	n	y	y	y	y	y	n	n	n	n	n	y	y	y	y	y	n	n	n	n	n	y	y	y	y	y	n	n	n	n	n	n	n	n	n	n
105	66-22-8	uracil	THF	n	N	n	n	n	n	y	y	y	y	n	y	y	y	y	y	y	y	y	y	y	n	n	n	n	n	y	y	y	y	n	y	y	y	y	y	y	n	y	y
106	121-33-5	vanillin	MeOH/ACN	n	N	n	n	n	y	y	y	y	y	n	n	n	n	n	y	y	n	y	y	n	n	n	n	n	y	y	y	y	y	n	n	n	n	n	n	n	n	n	n
107	224785-90-4	vardenafil	ACN	y	Y	y	y	y	n	y	y	y	n	y	y	y	y	y	n	y	n	y	y	y	y	y	y	y	n	n	n	n	n	y	y	y	y	y	y	y	y	y	y

Table S2: Molecular descriptors used in the study, calculated with CDK Descriptor Calculator (v.1.4.8).

Type and Class of Molecular descriptors	Individual descriptors (abbreviation)	Meaning
**ALOGP**<br>***Constitutional Descriptor***<br>***(atom additive logP and molar refractivity values; described by Ghose and Crippen)***	AlogP	Ghose-Crippen LogKow (octanol-water coefficient)
	AlogP2	Ghose-Crippen octanol water coefficient squared
	AMR	Ghose-Crippen molar refractivity
**APol**<br>***Electronic Descriptor***	Apol	sum of the atomic polarizabilities (including implicit hydrogens)
**AcidicGroupContent**<br>***Constitutional Descriptor***	nAcid	number of acidic groups
**BCUT**<br>***Hybrid Descriptor***<br>***(eigenvalue-based descriptor noted for its utility in chemical diversity described by Pearlman et al; a weighted version of the Burden matrix)***	BCUTw-1l	nhigh (number of highest eigenvalue) lowest atom weighted BCUTS
	BCUTw-1h	nlow (number of lowest eigenvalue) highest atom weighted BCUTS
	BCUTc-1l	nhigh (number of highest eigenvalue) lowest partial charge weighted BCUTS
	BCUTc-1h	nlow (number of lowest eigenvalue) highest partial charge weighted BCUTS
	BCUTp-1l	nhigh (number of highest eigenvalue) lowest polarizability weighted BCUTS
	BCUTp-1h	nlow (number of lowest eigenvalue) highest polarizability weighted BCUTS
**CPSA**<br>***Electronic and Geometrical Descriptor***<br>***(29 Charged Partial Surface Area Descriptors)***	PPSA-1	partial positive surface area; sum of surface area on positive parts of molecule
	PPSA-2	partial positive surface area * total positive charge on the molecule
	PPSA-3	charge weighted partial positive surface area
	PNSA-1	partial negative surface area; sum of surface area on negative parts of molecule
	PNSA-2	partial negative surface area * total negative charge on the molecule
	PNSA-3	charge weighted partial negative surface area
	DPSA-1	difference of PPSA-1 and PNSA-1
	DPSA-2	difference of FPSA-2 and PNSA-2
	DPSA-3	difference of PPSA-3 and PNSA-3
	FPSA-1	PPSA-1 / total molecular surface area
	FPSA-2	PPSA-2 / total molecular surface area
	FPSA-3	PPSA-3 / total molecular surface area
	FPNA-1	PNSA-1 / total molecular surface area
	FPNA-2	PNSA-2 / total molecular surface area
	FPNA-3	PNSA-3 / total molecular surface area
	WPSA-1	PPSA-1 * total molecular surface area / 1000
	WPSA-2	PPSA-2 * total molecular surface area / 1000
	WPSA-3	PPSA-3 * total molecular surface area / 1000
	WPNA-1	PNSA-1 * total molecular surface area / 1000
	WPNA-2	PNSA-2 * total molecular surface area / 1000
	WPNA-3	PNSA-3 * total molecular surface area / 1000
	RPCG	relative positive charge; most positive charge / total positive charge
	RNCG	relative negative charge; most negative charge / total negative charge

Type and Class of Molecular descriptors	Individual descriptors (abbreviation)	Meaning
	RPCS	relative positive charge surface area; most positive surface area * RPCG
	RNCS	relative negative charge surface area; most negative surface area * RNCG
	THSA	sum of solvent accessible surface areas of atoms with absolute value of partial charges < 0.2
	TPSA	sum of solvent accessible surface areas of atoms with absolute value of partial charges > 0.2
	RHSA	THSA / total molecular surface area
	RPSA	TPSA / total molecular surface area
**WHIM** *Hybrid Descriptor* ***(Weighted Holistic Invariant Molecular descriptors; based on a number of atom weightings)***	Wlambda1.unity;	directional descriptor; related to molecular size
	Wlambda2.unity	directional descriptor; related to molecular size
	Wlambda3.unity	directional descriptor; related to molecular size
	Wnu1.unity	directional descriptor; related to molecular shape
	Wnu2.unity	directional descriptor; related to molecular shape
	Weta1.unity	directional descriptor; related to density of the atoms distribution
	Weta2.unity	directional descriptor; related to density of the atoms distribution
	Weta3.unity	directional descriptor; related to density of the atoms distribution
	WT.unity	non-directional descriptor; related to linear contributions to the total molecular dimension
	WA.unity	non-directional descriptor; related to quadratic contributions to the total molecular dimension
	WV.unity	non-directional descriptor; contains also the third-order term;
	WK.unity	non-directional descriptor; molecular shape
	WD.unity	non-directional descriptor; the total molecular density
**MDE** *Topological Descriptor* (***Molecular Distance Edge Descriptors for C, O, and N***)	MDEC-11	molecular distance edge between all primary carbons
	MDEC-12	molecular distance edge between all primary and secondary carbons
	MDEC-13	molecular distance edge between all primary and tertiary carbons
	MDEC-14	molecular distance edge between all primary and quaternary carbons
	MDEC-22	molecular distance edge between all secondary carbons
	MDEC-23	molecular distance edge between all secondary and tertiary carbons
	MDEC-24	molecular distance edge between all secondary and quaternary carbons
	MDEC-33	molecular distance edge between all tertiary carbons
	MDEC-34	molecular distance edge between all tertiary and quaternary carbons
	MDEC-44	molecular distance edge between all quaternary carbons
	MDEO-11	molecular distance edge between all primary oxygens
	MDEO-12	molecular distance edge between all primary and secondary oxygens
	MDEO-22	molecular distance edge between all secondary oxygens
	MDEN-12	molecular distance edge between all primary and secondary nitrogens
	MDEN-13	molecular distance edge between all primary and tertiary nitrogens
	MDEN-22	molecular distance edge between all secondary nitrogens
	MDEN-23	molecular distance edge between all secondary and tertiary nitrogens
	MDEN-33	molecular distance edge between all tertiary nitrogens
**AromaticAtomsCount** ***Constitutional Descriptor***	naAromAtom	number of aromatic atoms in an atom container

Type and Class of Molecular descriptors	Individual descriptors (abbreviation)	Meaning
**AromaticBonsCount**<br>***Constitutional Descriptor***	nAromBond	number of aromatic atoms in an AtomContainer; based on the number of aromatic bounds
**AtomCount**<br>***Constitutional Descriptor***	nAtom	number of atoms of a certain element type
**AutocorrelationCharge**<br>***Topological Descriptor***<br>***(the Moreau-Broto autocorrelation descriptors using partial charges)***	ATSc1	ATS autocorrelation descriptor, weighted by charges
	ATSc2	ATS autocorrelation descriptor, weighted by charges
	ATSc3	ATS autocorrelation descriptor, weighted by charges
	ATSc4	ATS autocorrelation descriptor, weighted by charges
	ATSc5	ATS autocorrelation descriptor, weighted by charges
**AutocorrelationMass**<br>***Topological Descriptor***<br>***(the Moreau-Broto autocorrelation descriptors using atomic weight)***	ATSm1	ATS autocorrelation descriptor, weighted by scaled atomic mass
	ATSm2	ATS autocorrelation descriptor, weighted by scaled atomic mass
	ATSm3	ATS autocorrelation descriptor, weighted by scaled atomic mass
	ATSm4	ATS autocorrelation descriptor, weighted by scaled atomic mass
	ATSm5	ATS autocorrelation descriptor, weighted by scaled atomic mass
**AutocorrelationPolarizability**<br>***Topological Descriptor***<br>***(the Moreau-Broto autocorrelation descriptors using polarizability)***	ATSp1	ATS autocorrelation descriptor, weighted by polarizability
	ATSp2	ATS autocorrelation descriptor, weighted by polarizability
	ATSp3	ATS autocorrelation descriptor, weighted by polarizability
	ATSp4	ATS autocorrelation descriptor, weighted by polarizability
	ATSp5	ATS autocorrelation descriptor, weighted by polarizability
**BPol**<br>***Electronic Descriptor***	Bpol	sum of the absolute value of the difference between atomic polarizabilities of all bonded atoms in the molecule (including implicit hydrogens)
**BasicGroupCount**<br>***Constitutional Descriptor***	nBase	number of basic groups
**BondCount**<br>***Constitutional Descriptor***	nBx	single value with name nBX where X can be s(single bond), d (double), t (triple), a (aromatic), “”(all)
**CarbonTypes**<br>***Topological Descriptor***<br>***(carbon connectivity in the terms of hybridization)***	C1SP1	triply bound carbon bound to one other carbon
	C1SP2	triply bound carbon bound to two other carbons
	C2SP2	doubly bound carbon bound to two other carbons
	C3SP2	doubly bound carbon bound to three other carbons
	C1SP3	singly bound carbon bound to one other carbon
	C2SP3	singly bound carbon bound to two other carbons
	C3SP3	singly bound carbon bound to three other carbons
	C4SP3	singly bound carbon bound to four other carbons
**ChiChain**<br>***Topological Descriptor***<br>***(evaluates the Kier & Hall Chi chain indices of orders 3,4,5 and 6; type of chain)***	SCH-3	simple chain, order 3
	SCH-4	simple chain, order 4
	SCH-5	simple chain, order 5
	SCH-6	simple chain, order 6
	SCH-7	simple chain, order 7
	VCH-3	valence chain, order 3

Type and Class of Molecular descriptors	Individual descriptors (abbreviation)	Meaning
	VCH-4	valence chain, order 4
	VCH-5	valence chain, order 5
	VCH-6	valence chain, order 6
	VCH-7	valence chain, order 7
**ChiCluster** *Topological Descriptor* ***(evaluates the Kier & Hall Chi cluster indices of orders 3,4,5, and 6)***	SC-3	simple cluster, order 3
	SC-4	simple cluster, order 4
	SC-5	simple cluster, order 5
	SC-6	simple cluster, order 6
	VC-3	valence cluster, order 3
	VC-4	valence cluster, order 4
	VC-5	valence cluster, order 5
	VC-6	valence cluster, order 6
**ChiPathCluster** *Topological Descriptor* ***(evaluates the Kier & Hall Chi path cluster indices of orders 4,5, and 6)***	SPC-4	simple path cluster, order 4
	SPC-5	simple path cluster, order 5
	SPC-6	simple path cluster, order 6
	VPC-4	valence path cluster, order 4
	VPC-5	valence path cluster, order 5
	VPC-6	valence path cluster, order 6
**ChiPath** *Topological Descriptor* ***(evaluates the Kier & Hall Chi path indices of orders 0,1,2,3,4,5,6 and 7)***	SP-0	simple path, order 0
	SP-1	simple path, order 1
	SP-2	simple path, order 2
	SP-3	simple path, order 3
	SP-4	simple path, order 4
	SP-5	simple path, order 5
	SP-6	simple path, order 6
	SP-7	simple path, order 7
	VP-0	valence path, order 0
	VP-1	valence path, order 1
	VP-2	valence path, order 2
	VP-3	valence path, order 3
	VP-4	valence path, order 4
	VP-5	valence path, order 5
	VP-6	valence path, order 6
	VP-7	valence path, order 7
**EccentricConnectivityIndex** *Topological Descriptor*	ECCEN	combining distance and adjacency information
**FMF** *Topological Descriptor*	FMF	ratio of heavy atoms in the framework to the total number of heavy atoms in the molecule; characterize the complexity of the molecule

Type and Class of Molecular descriptors	Individual descriptors (abbreviation)	Meaning
**FragmentComplexity**<br>***Topological Descriptor***	fragC	Complexity of a system; C=abs(B^2-A^2+A)+H/100 where C=complexity, A=number of non-hydrogen atoms, B=number of bonds and H=number of heteroatoms
**GravitationalIndex**<br>***Geometrical Descriptor***<br>***(mass distribution of the molecule)***	GRAV-1	gravitational index of heavy atoms
	GRAV-2	square root of gravitational index of heavy atoms
	GRAV-3	cube root of gravitational index of heavy atoms
	GRAVH-1	gravitational index - hydrogens included
	GRAVH-2	square root of hydrogen-included gravitational index
	GRAVH-3	cube root of hydrogen-included gravitational index
	GRAV-4	grav1 for all pairs of atoms (not just bonded pairs)
	GRAV-5	grav2 for all pairs of atoms (not just bonded pairs)
	GRAV-6	grav3 for all pairs of atoms (not just bonded pairs)
**HBondAcceptorCount**<br>***Electronic Descriptor***	nHBAcc	the number of H bond acceptors using a slightly simplified version of the PHACIR atom types. The groups counted as acceptors: (i) Any O where the formal charge of the oxygen is non-positive (i.e. formal charge ≤ 0) except an aromatic ether O and an O that is adjacent to a N; (ii) Any N where the formal charge of the N is non-positive (i.e. formal charge ≤ 0) except a N that is adjacent to an O
**HBondDonorCount**<br>***Electronic Descriptor***	nHBDon	number of H bond donors using a slightly simplified version of the PHACIR atom types. The groups counted as donors: (i) Any-OH where the formal charge of the O is non-negative (i.e. formal charge ≥ 0); (ii) Any-NH where the formal charge of the N is non-negative (i.e. formal charge ≥ 0)
**HybridizationRatio**<br>***Topologocial Descriptor***	HybRatio	the fraction of sp3 carbons to sp2 carbons; complexity of the molecule
**KierHallSmarts**<br>***Topological Descriptor***<br>***(it counts the number of occurrences of the E-state fragments;***<br>***- single bond; =double bond; # triple bond; : aromatic bond)***	khs.sCH3	count of atom-type E-state: $-CH_3$
	khs.ssCH2	count of atom-type E-state: $-CH_2-$
	khs.dsCH	count of atom-type E-state: =CH-
	khs.aaCH	count of atom-type E-state: :CH:
	khs.sssCH	count of atom-type E-state: >CH-
	khs.tsC	count of atom-type E-state: #C-
	khs.dssC	count of atom-type E-state: =C<
	khs.aasC	count of atom-type E-state: :C:-
	khs.aaaC	count of atom-type E-state: ::C:
	khs.ssssC	count of atom-type E-state: >C<
	khs.sNH2	count of atom-type E-state: $-NH_2$
	khs.ssNH	count of atom-type E-state: $-NH_2-^{+}$
	khs.aaNH	count of atom-type E-state: :NH:
	khs.tN	count of atom-type E-state: #N
	khs.aaN	count of atom-type E-state: :N:
	khs.sssN	count of atom-type E-state: >N-
	khs.aasN	count of atom-type E-state: :N:-

Type and Class of Molecular descriptors	Individual descriptors (abbreviation)	Meaning
	khs.sOH	count of atom-type E-state: -OH
	khs.dO	count of atom-type E-state: =O
	khs.ssO	count of atom-type E-state: -O-
	khs.aaO	count of atom-type E-state: :O:
	khs.sF	count of atom-type E-state: -F
	khs.dsssP	count of atom-type E-state: ->P=
	khs.dS	count of atom-type E-state: =S
	khs.ssS	count of atom-type E-state: -S-
	khs.aaS	count of atom-type E-state: :S:
	khs.ddssS	count of atom-type E-state: >S==
	khs.sCl	count of atom-type E-state: -Cl
	khs.sBr	count of atom-type E-state: -Br
**KappaShapeIndices**	Kier1	first kappa shape index
Topological Descriptor	Kier2	second kappa shape index
(Kier and Hall kappa molecular shape indices compare the molecular graph with minimal and maximal molecular graphs)	Kier3	third kappa shape index
	MOMI-X	moment of inertia along X axis
**MomentOfInertia**	MOMI-Y	moment of inertia along Y axis
Geometrical Descriptor	MOMI-Z	moment of inertia along Z axis
(principal moment of inertia, their ratios,	MOMI-XY	ratio X/Y
and radius of gyration; characterize the	MOMI-XZ	ratio X/Z
mass distribution of a molecule)	MOMI-YZ	ratio Y/Z
	MOMI-R	radius of the gyration of the molecule
**WeightedPath**	WTPT-1	molecular ID
Topological Descriptor	WTPT-2	molecular ID / number of atoms
(The weighted path (molecular ID)	WTPT-3	sum of path lengths starting from heteroatoms
descriptors described by Randic. They	WTPT-4	sum of path lengths starting from oxygens
characterize molecular branching.)	WTPT-5	sum of path lengths starting from nitrogens
**WienerNumbers**	WPATH	weiner path number
Topological Descriptor (described by Randic, characterize molecular branching)	WPOL	weiner polarity number
**LargestChain** ***Constitutional Descriptor***	nAtomLC	number of atoms in the largest chain
**LargestPiSystem** ***Constitutional Descriptor***	nAtomP	number of atoms in the largest pi system
**LongestAliphaticChain**	nAtomLAC	number of atoms in the longest aliphatic chain

Type and Class of Molecular descriptors	Individual descriptors (abbreviation)	Meaning
Constitutional Descriptor		
**PetitjeanNumber** ***Topological Descriptor***	PetitjeanNuber	the eccentricity of a vertex corresponds to the distance from that vertex to the most remote vertex in the graph. The distance is obtained from the distance matrix as the count of edges between the two vertices. If r(i) is the largest matrix entry in row i of the distance matrix D, then the radius is defined as the smallest of the r(i). The graph diameter D is defined as the largest vertex eccentricity in the graph
**MannholdLogP** ***Constitutional Descriptor***	MlogP	prediction of logP based on the number of carbon and hetero atoms
**PetitjeanShapeIndex** ***Topological/Geometrical Descriptor (anisotropy of molecule)***	topoShape<br>geomShape	topological shape index<br>geometric shape index
**RuleOfFive** ***Constitutional Descriptor***	LipinskiFailures	number failures of the Lipinski's Rule Of 5
**TPSA** ***Topological/Electronic Descriptor***	TopoPSA	topological polar surface area based on fragment contributions
**VABC** ***Constitutional Descriptor***	VABC	values derived from the van der Waals Volume as a Sum of Atomic and Bond Contributions (VABC)
**VAdjMa** ***Topological Descriptor***	VadjMat	Vertex adjacency information (magnitude): 1 + log2 m where m is the number of heavy-heavy bonds
**Weight** ***Constitutional Descriptor***	MW	based on the weight of atoms of a certain element type
**XLogP** ***Constitutional Descriptor***	XlogP	prediction of logP based on the atom-type method
**ZagrebIndex** ***Topological Descriptor***	Zagreb	the sum of the squares of atom degree over all heavy atoms i
**RotatableBondsCount** ***Constitutional Descriptor***	nRotB	number of rotatable bonds is given by the SMARTS specified by Daylight
Other Constitutional Descriptors	tpsaEfficiency<br>nSmallRings<br>nAromRings<br>nRingBlocks<br>nAromBlocks<br>nRings3<br>nRings4<br>nRings5<br>nRings6<br>nRings7	Polar surface area expressed as a ratio to molecular size<br>Number of small rings from size 3 to 9<br>Number of aromatic rings<br>Total number of distinct ring blocks<br>Total number of “aromatically connected components”<br>individual breakdown of small ring, size 3<br>individual breakdown of small ring, size 4<br>individual breakdown of small ring, size 5<br>individual breakdown of small ring, size 6<br>individual breakdown of small ring, size 7

Chromatographic conditions.

The reference standards were divided into several working mixtures according to their respective m/z and retention times to avoid coelution and to allow correct peak area determination. The concentration of the reference standards in each working mixture was 1 µg/mL. ACN was used for the dilution. The separation was carried out on the Torus Diol column (100 × 3 mm, 1.7 µm; Waters) using a generic gradient method with a mobile phase consisting of CO_2 (A) and organic modifier (B) at a flow rate of 1.5 mL/min: 2% B for 1 min, 2 – 45% B in 1.0 – 3.0 min, followed by 1 min isocratic step at 45% B and 1.5 min equilibration at initial conditions. Two different organic modifiers were used: (i) MeOH and (ii) MeOH + 10 mM NH_3. The column temperature was maintained at 40 °C and BPR at 13 MPa (≈ 1885 psi). 2 µL of the standard solution were injected into the system using a partial loop with needle overfill injection mode. The autosampler temperature was 10 °C. Methanol was used as the weak and strong wash solvent.

Equations S1. *Equations used for the calculation of measured-adjusted responses adjusted from Plachka et al.*[1]

The QC-corrected responses were recalculated for 100 µL MeOH entering the MS source. The SFC-MS coupling used in this study was facilitated by a Waters interface consisting of two T-unions. In the first T-union, the eluent from the column was mixed with makeup solvent. The flow was then split between the BPR regulator and the MS inlet in the second T-union. The splitting ratio is defined by several parameters, including the viscosity of the CO_2/methanol mixture and the used capillaries. Due to the different solvent composition during gradient and thus, different splitting ratios, a different volume of organic solvent enters the MS at each retention time. The calculated volume of organic solvent entering the MS ionization sources in this study ranged from 70 to 320 µL. Therefore, 100 µL was selected as a comparable value within this range.

According to available literature[1-4], the calculations for the adjustment of measured MS responses to 100 µL methanol entering MS should be as follows:

As described by Grand-Guillaume Perrenoud *et al.*[2]*,* when performing measurements with methanol as an organic modifier of CO_2-based mobile phase without any specific SFC-MS interface, i.e., with no makeup solvent and no split, the MS response at each retention time (t) ($R_{ESI, MeOH}$) is dependent on the sensitivity (S_{ESI}) and concentration of the analyte ($C_{chrom, (t)}$) (1).

(1) $$R_{ESI,\,MeOH\,(t)} = S_{ESI} * C_{chrom\,(t)}$$

When the SFC-MS interface is used, the analyte is firstly diluted by a factor D_F in the first T-union due to the addition of a makeup solvent (2).

(2) $$D_F = \frac{F_{tot}}{F_{SFC}}$$

Where F_{tot} is the total flow rate, i.e., both the SFC and sheath pump and F_{SFC} is the flow rate only from the SFC pump. In the second T-union, CO_2 is depressurized in the restriction capillary between the column outlet and MS inlet, and the analyte is concentrated by a factor C_F (3).

(3) $$C_F = \frac{F_{tot}}{F_{MeOH,MS}}$$

Where $F_{MeOH,\,MS}$ is the total volume of methanol entering the MS ionization source. This means that the final MS response is affected by a coefficient λ (4 and 5).

(4) $$\lambda_{ESI} = C_F/D_F$$

(5) $$R_{ESI(t)} = R_{ESI,MeOH(t)} * \lambda_{ESI}$$

For calculations, it is necessary to know the total volume of methanol entering the MS at each retention time $F_{MeOH,\,MS}$ (6).

(6) $$F_{MeOH,\,MS} = F_{MeOH,tot} * \frac{(128 * \eta * R_{BPR} * F_{tot} + \pi\ * \Delta P_{BPR})}{128 * \eta * (R_{MS} + R_{BPR}) * F_{tot}}$$

Where $F_{MeOH, tot}$ is the total flow of methanol mobile phase + make-up solvent, η is the viscosity of the CO_2/MeOH mixture at specific temperature and pressure, ΔP_{BPR} is the back-pressure, i.e., system pressure, and R_{BPR} and R_{MS} correspond to the ratio between length and diameter of the capillary leading form the second T-union to the back-pressure regulator and MS, respectively. The system pressure recorded for each analysis was used for the particular calculation. The calculation of viscosity of pure CO_2 at specific temperature and pressure is then based on correlations proposed by Ouyang[5]:

(7) $\eta\,(P,T) = \sum_{i=0}^{4} c_i P^i$

Where P is the pressure, T is the temperature, and c_i is the temperature-dependent coefficient that can be written as follows:

(8) $c_i(T) = \sum_{j=0}^{4} d_i T^i$

Where d_i is the model coefficient. It is necessary to use a correlation of second-order between viscosity and methanol content η to calculate the viscosity of the CO_2/methanol mixture. It can be done based on the experimentally determined data published by Sih *et al.*[4]:

(9) $\eta(X) = a_0 + a_1 X + a_2 X$

Where X is the methanol fraction of the mobile phase and a_i is the correlation coefficient.

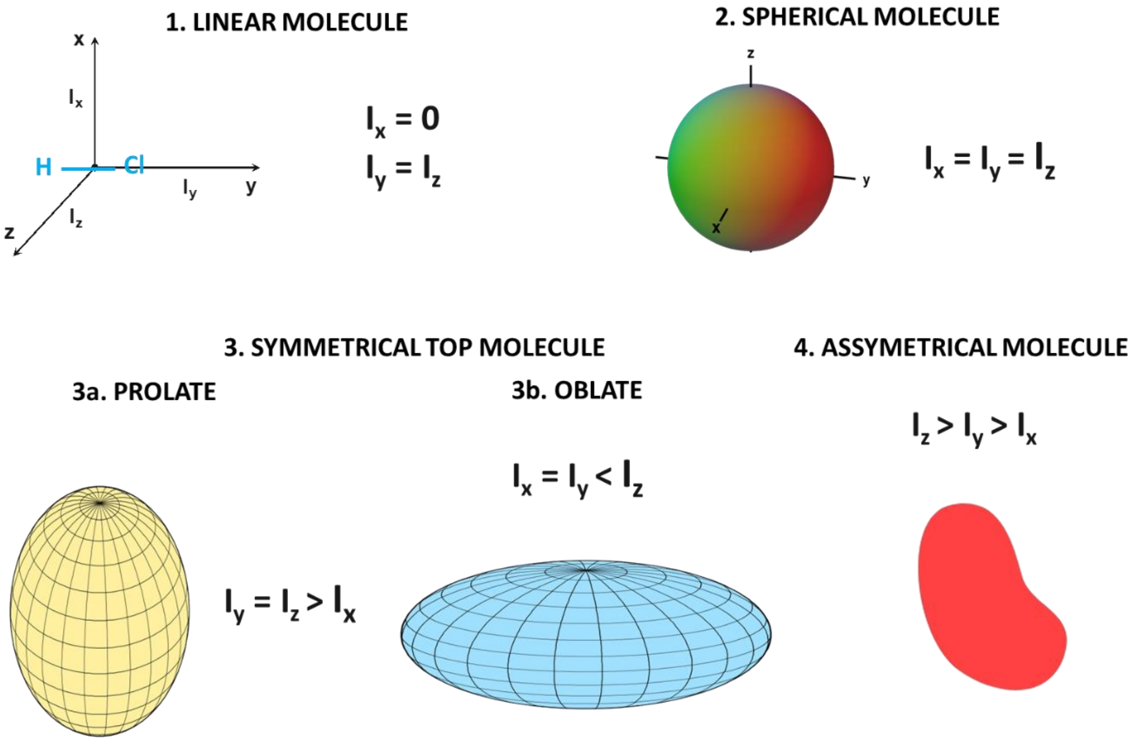

Figure S1. Description and general representation of the moment of inertia (MOMI) molecular descriptor.

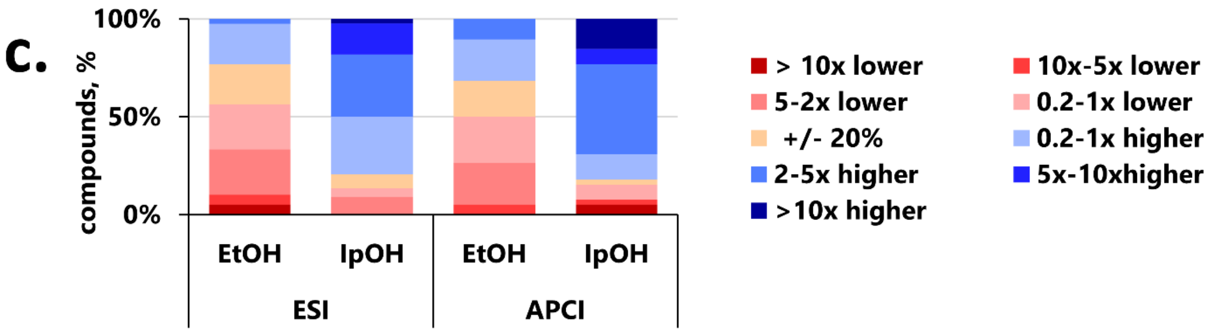

Figure S2.The effect of ethanol (EtOH) and isopropanol (IpOH) as makeup solvents on MS responses compared to MS responses obtained with MeOH.

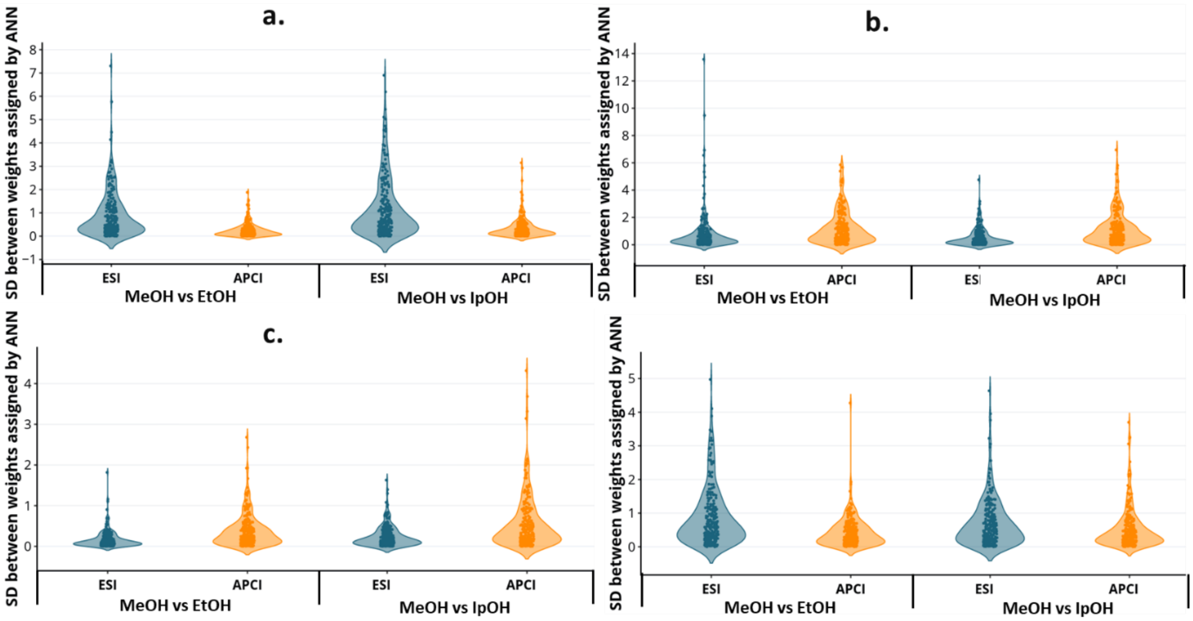

Figure S3. Violin plots summarizing standard deviations (SD) between weights assigned by ANN to the molecular descriptors when using ethanol (EtOH) and propan-2-ol (IpOH) instead of methanol (MeOH) as makeup solvent. Conditions: a – MeOH as organic solvent, positive ionization mode, b – MeOH as organic modifier, negative ionization mode, c – 10 mmol/L NH_3 in MeOH, positive ionization mode, d – 10 mmol/L NH_3 in MeOH, negative positive mode. Violin plots combine a box plot and a rotated density plot. They can thus graphically represent the distribution of the data population where the shape of the violin plot corresponds to the density estimate of the data points, i.e., regions with a higher frequency of finding particular values appear wider in the violin plot.

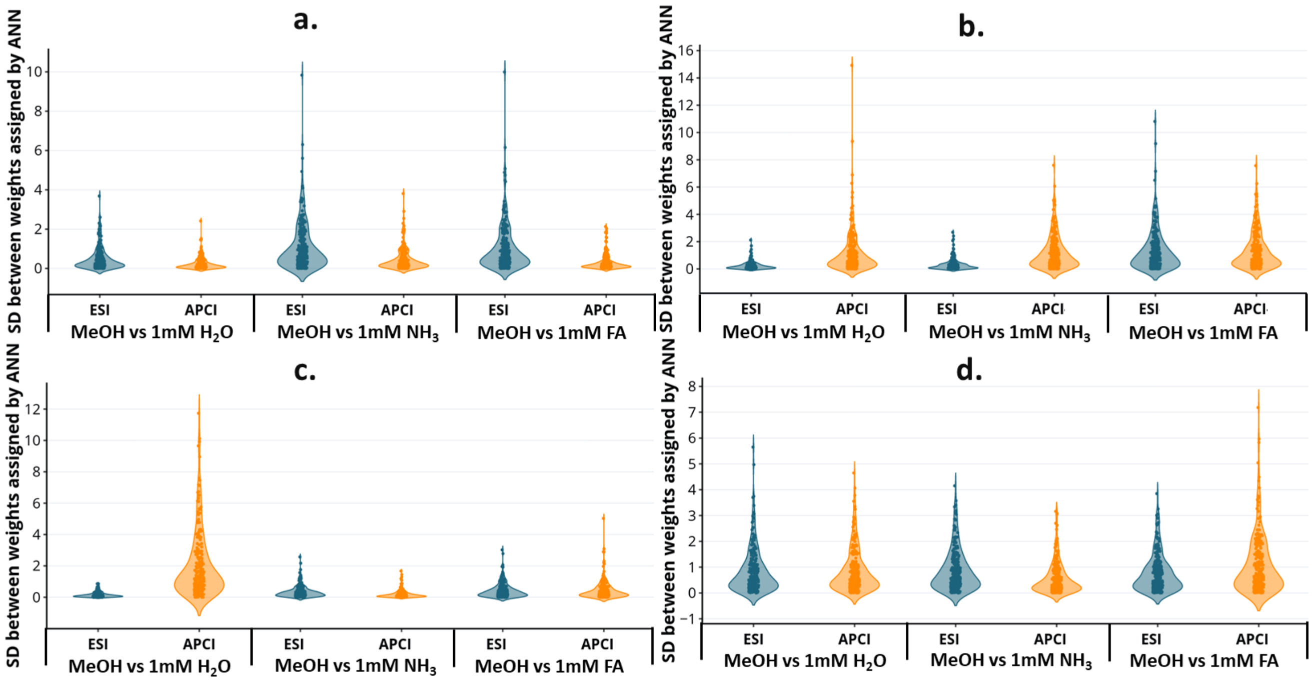

Figure S4. Violin plots summarizing standard deviations (SD) between weights assigned by ANN to the molecular descriptors when using 1mM H_2O in methanol, 1mM NH_3 in methanol, and 1mM formic acid (FA) instead of methanol (MeOH) as makeup solvent. Conditions: a – MeOH as organic solvent, positive ionization mode, b – MeOH as organic modifier, negative ionization mode, c – 10 mmol/L NH_3 in MeOH, positive ionization mode, d – 10 mmol/L NH_3 in MeOH, negative positive mode.

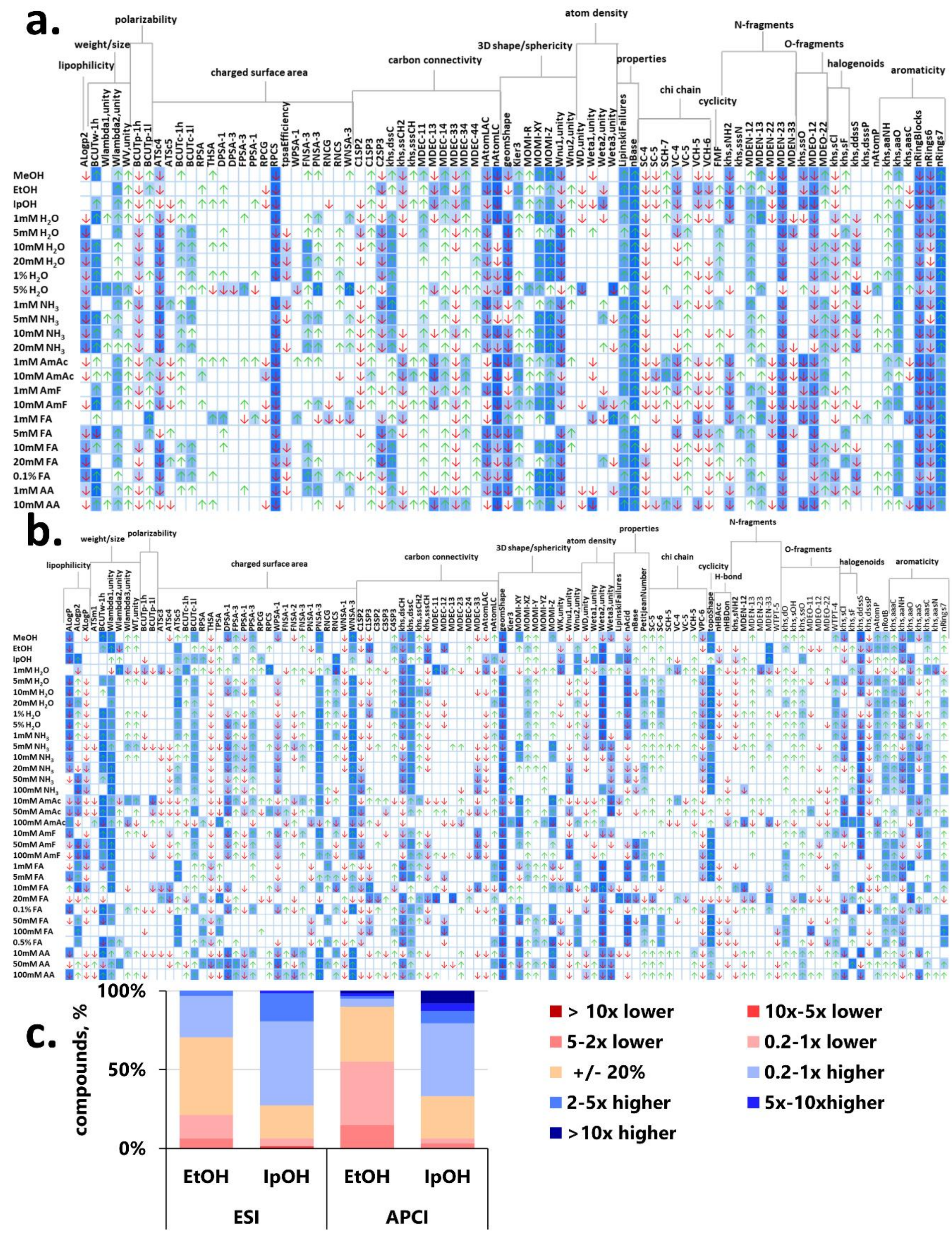

Figure S5. Key molecular descriptors affecting ionization in ESI+ (a) and APCI+ (b) when using 10 mmol/L NH_3 in MeOH as organic modifier. The shade of blue in the heatmaps corresponds to the ranking of the molecular descriptor for each makeup solvent composition (ranking 1 = the highest absolute ANN-assigned weight = the darkest blue). ↑- increasing effect on ionization, ↓-decreasing effect on ionization. (c) The effect of ethanol (EtOH) and isopropanol (IpOH) as makeup solvents on MS responses compared to MS responses obtained using MeOH.

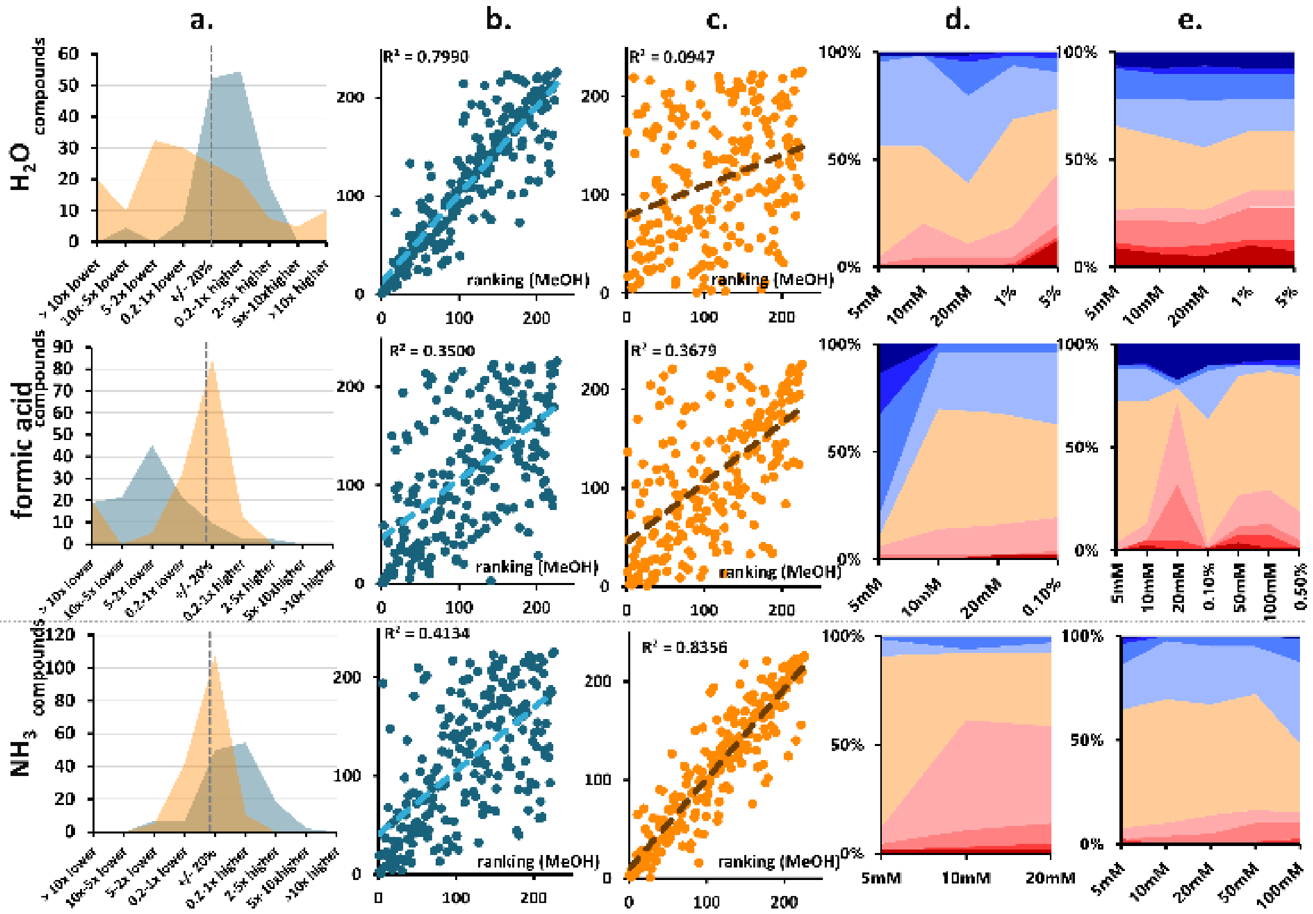

Figure S6. Effect of 1 mmol/L additive in makeup solvent compared to pure methanol as makeup solvent on MS responses (a) and a comparison of rankings of molecular descriptors based on ANN-assigned weights (b, c) for ESI$^+$ (blue) and APCI$^+$ (orange). Effect of increasing additive concentration on MS responses in ESI$^+$ (d) and APCI$^+$ (e) compared to MS responses obtained using 1 mmol/L additive in methanol. Over 10-fold lower (dark red), 10-5-fold lower (red), 5-2-fold lower (dark pink), 0.2-1-fold lower (pink), within +/- 20% (orange), 0.2-1-fold higher (the lightest blue), 2-5-fold higher (light blue), 5-10-fold higher (blue), over 10-fold higher (dark blue). All results were obtained using 10 mmol/L ammonia in methanol as the organic modifier.

Table S3. Correlation coefficients between molecular descriptor weights assigned by ANN based on makeup solvents with various concentrations of additives compared to 1 mM.

		MeOH as organic modifier				10 mM NH_3 in MeOH as organic modifier			
		ESI^+	$APCI^+$	ESI^-	$APCI^-$	ESI^+	$APCI^+$	ESI^-	$APCI^-$
vs 1 mM H_2O	5mM H_2O	0.8147	0.8423	0.9703	0.7370	0.8096	0.2083	0.8463	0.7216
	10mM H_2O	0.8673	0.7721	0.9606	0.6122	0.8614	0.1912	0.6687	0.6112
	20mM H_2O	0.8890	0.8743	0.8535	0.5332	0.8518	0.2424	0.6741	0.7742
	1% H_2O	0.8527	0.7282	0.2332	0.3445	0.8743	0.1940	0.6651	0.7365
	5% H_2O	0.7412	0.7580	0.3597	0.6801	0.7698	0.1790	0.5733	0.6768
vs 1 mM FA	5mM FA	0.9675	0.8925	0.9523	0.7993	0.6028	0.9654	0.8667	0.8708
	10mM FA	0.9811	0.9023	0.9988	0.9335	0.5944	0.0147	0.8012	0.5086
	20mM FA	0.9693	0.3619	0.9789	0.4794	0.4916	0.2010	0.7920	0.5991
	0.1% FA	0.8811	0.8816	0.9808	0.7318	0.4988	0.5895	0.8630	0.7483
	50mM FA		0.9200		0.9055		0.9403		0.6539
	100mM FA		0.9154		0.8307		0.8695		0.9582
	0.5% FA		0.9529		0.9172		0.9439		0.8994
	1mM AA	0.9782		0.8210		0.5405		0.8962	
	10mM AA	0.9556	0.8596	0.9663	0.5940	0.7184	0.4320	0.8112	0.8534
	50mM AA		0.8057		0.5868		0.7265		0.6582
	100mM AA		0.8112		0.4598		0.5019		0.5464
vs 1 mM NH_3	5mM NH_3	0.9899	0.9832	0.7351	0.9268	0.9330	0.8663	0.8579	0.8944
	10mM NH_3	0.7637	0.9872	0.9812	0.8028	0.9082	0.9367	0.8607	0.9014
	20mM NH_3	0.8088	0.9740	0.7919	0.5768	0.9135	0.8999	0.7954	0.8840
	50mM NH_3		0.8898		0.5763		0.7982		0.7589
	100mM NH_3		0.9333		0.6643		0.5978		0.2546
	1mM AmAc	0.9948		0.9522		0.7206		0.8870	
	10mM AmAc	0.7223	0.9221	0.1286	0.6680	0.6666	0.5669	0.1813	0.6235
	50mM AmAc		0.9242		0.4083		0.6961		0.5653
	100mM AmAc		0.9169		0.5941		-0.2376		0.5542
	1mM AmF	0.9672		0.7315		0.8300		0.8093	
	10mM AmF	0.9596	0.5577	0.9191	0.8585	0.7151	0.8648	0.7647	0.6842
	50mM AmF		0.9228		0.5835		0.7297		0.3652
	100mM AmF		0.8968		0.5191		0.7137		0.6491

Reference

(1) Plachká, K.; Gazárková, T. á.; Škop, J.; Guillarme, D.; Svec, F.; Nováková, L. *Analytical Chemistry* **2022**, *94* (11), 4841-4849, DOI: 10.1021/acs.analchem.2c00154

(2) Grand-Guillaume Perrenoud, A.; Hamman, C.; Goel, M.; Veuthey, J.-L.; Guillarme, D.; Fekete, S. *Journal of Chromatography A* **2013**, *1314*, 288-297, DOI: https://doi.org/10.1016/j.chroma.2013.09.039

(3) Ouyang, L.-B. *The Open Petroleum Engineering Journal* **2011**, *5*, DOI: 10.2174/1874834101104010013

(4) Sih, R.; Dehghani, F.; Foster, N. R. *The Journal of Supercritical Fluids* **2007**, *41* (1), 148-157, DOI: https://doi.org/10.1016/j.supflu.2006.09.002

(5) Ouyang, L.-B. *The Open Petroleum Engineering Journal* **2011**, *12* (4), 13-21, DOI: 10.2174/1874834101104010013
