# Supplementary material for: Artificial Neural Networks: An Innovative Approach Used for Elucidation of Ionization Processes in Supercritical Fluid Chromatography-Mass Spectrometry
Source: Anal Chem. 2025 May 10;97(19):10252–63. doi: 10.1021/acs.analchem.5c00152 (PMC12096348; doi:10.1021/acs.analchem.5c00152)
Supplement: Supplementary file 2 [file ac5c00152_si_002.pdf]

Supporting Information 2: Artificial neural networks: an innovative approach used for elucidation of ionization processes in supercritical fluid chromatography-mass spectrometry

Kateřina Plachká[1], Veronika Pilařová[1], Taťána Gazárková[1], Jean-Christophe Garrigues[2], František Švec[1], Lucie Nováková[1*]

[1] Department of Analytical Chemistry, Faculty of Pharmacy in Hradec Králové, Charles University, Akademika Heyrovského 1203/8, 50003 Hradec Králové, Czechia

[2] SOFTMAT (IMRCP) Laboratory, SMODD Team, CNRS, Toulouse III Paul Sabatier University, 31400 Toulouse, France

* Email: nol@email.cz (Lucie Nováková)

Table of Content

Results and discussion: Negative ionization mode

Elucidation of ionization process in SFC-MS using methanol as organic modifier. ***Negative ionization mode.*** *Ionization mechanisms using methanol as makeup solvent.* Comparison of ANN-assigned weights of the key molecular descriptors when measured in negative ionization mode (Supporting Information (SI) 2 Figure S1a,b) confirmed the results from Figure 1 with quite similar ionization behavior in both ESI$^-$ and APCI$^-$.

The importance of charge surface area in negative mode was confirmed by the high weights assigned to BCUTc-1h (SI 2 Figure S1a,b). Ionization in ESI$^-$ and APCI$^-$ was more efficient for molecules with high values of DPSA-1, i.e., molecules with a predominantly partial positive surface area, FNSA-2, related to the negative charge (SI 1 Table S2), and RPCG, i.e., the relative positive charge. The ANN results show that molecules with a predominantly positive charge (high DPSA-1) and low negative charge (high FNSA-2) will have better ionization efficiency in ESI$^-$ and APCI$^-$. The decreasing effect of partial negative charge on ionization efficiency was further confirmed by the significant negative weights assigned by ANN to PNSA-1 and FNSA-1 (SI 2 Figure S1a,b). RPCG shows that molecules with low total positive charge and high values of the most positive charge were more efficiently ionized in both ESI$^-$ and APCI$^-$. Contrarily, the negative effect was attributed to the relative positive charge surface area (RPCS) with high values for molecules with high positive charge localized on a large part of the molecule. This shows that the localization of the positive partial charge on the surface area plays an important role in the ionization mechanism. The most significant difference between ESI$^-$ and APCI$^-$ when evaluating the charge state was observed for the relative negative charge (RNCG). A low total negative charge with a high negative charge localized on a small part of the molecule (high RNCG and low RNCS) was beneficial in APCI$^-$.

The atom distribution played an important role in the ionization efficiency, as shown by the WHIM descriptors Weta1.unity, Weta3.unity, and WD.unity. Ionization in ESI$^-$ and APCI$^-$ increased with Br- and F- substitutions confirmed by khs.sF and khs.sBr (SI 2 Figure S1a,b), while chlorine substitution had no significant effect. The presence of secondary amines -NH- (khs.ssNH) increased the ionization efficiency in ESI$^-$, a larger molecular distance between secondary and tertiary amines was beneficial (MDEN-23). E-state fragments containing N atoms (N-fragment) had only a marginal effect on ionization in APCI$^-$. Aromatically bonded oxygen (khs.aaO) decreased ionization in both ion sources. The other molecular descriptors related to e-state fragments containing oxygen atoms (O-fragments) had no significant effect on ionization in ESI$^-$. In contrast, =O and -OH groups (khs.dO, khs.sOH) increased ionization in APCI$^-$, whereas -O- groups (khs.ssO) decreased ionization. A larger molecular distance between two primary and two secondary oxygen groups increased ionization (MDEO-11, MDEO-22), while a large distance edge between primary and secondary oxygens decreases ionization (MDEO-12). The small effect of N- and O- fragments on ionization in ESI$^-$ corresponds to the significant negative weights assigned by ANN to nAcid and nBase. Thus, compounds without acidic and alkaline functional groups ionized more efficiently. In APCI$^-$, the possibility of H-bond formation was more important. nHBDon groups increased ionization whereas nHBAcc decreased ionization.

The molecular size (BCUTw-1h) decreased the ionization efficiency also in ESI$^-$ and APCI$^-$. Wlambda1, Wlambda2, and Wlambda3 related to the molecular size showed a strong effect on ionization that varied between the ionization sources. AlogP, Ghose-Crippen-Viswanadhan octanol-water partition coefficient, and ATSm1, i.e., autocorrelation descriptor of mass, were more pronounced for ESI$^-$ and APCI$^-$. The positive weight of AlogP and XLogP combined with the negative weight of AlogP2 clearly shows that lipophilic compounds were more easily ionized in both ion sources but topological indices and intramolecular bonding further increasing lipophilicity were not beneficial.

Top symmetric molecules (high MOMI-Y and low MOMI-XY) were ionized more efficiently in ESI$^-$ than spherical compounds (high MOMI-Z). Linear compounds exhibited higher ionization efficiency in APCI$^-$ (low MOMI-Y, MOMI-Z, MOMI-XY, geomShape). The FMF, which characterizes the 2D structure and molecular complexity significantly increased ionization in APCI$^-$. Since the cyclic part of molecules usually results in more planar structure of the compounds, the opposite behavior of geomShape and FMF corresponds to the same behavior. Low polarizability (BCUTp-1l) significantly increased ionization in APCI$^-$.

The effect of alcohol type. The use of EtOH and IpOH was detrimental in ESI$^-$, with >70% of compounds having lower MS responses compared to MeOH as a makeup solvent, while mostly higher MS responses were observed in APCI$^-$ (SI 2 Figure S1c). These changes correspond to higher SD between molecular descriptor weights obtained using each alcohol (SI 1 Figure S3b).

The type of alcohol in ESI$^-$ mainly affected the MOMI and WHIM descriptors (SI 2 Figure S1a), suggesting a change in drop formation and evaporation. EtOH should be used as a makeup solvent in ESI$^-$ when analyzing compounds with high values of AlogP2. Carbon connectivity and hybridization played a more important role in ionization using EtOH and IpOH compared to MeOH in ESI$^-$. The 3D shape affected the ionization to a greater extent using IpOH (SI 2 Figure S1a). The alcohol type also mainly affected the WHIM and MOMI descriptors in APCI$^-$. Furthermore, the negative effect of secondary oxygen (khs.ssO) and the positive effect of lipophilicity (ALogP) were mitigated using EtOH and IpOH (SI 2 Figure S1b). Additionally, EtOH should be preferred in APCI$^-$ for easier ionization of compounds with high RNCS values.

The effect of additive. Water. The effect in negative mode was more dependent on analyte properties compared to the positive ionization mode Both large increases and decreases in MS responses were observed. The addition of 1 mmol/L H_2O slightly decreased MS responses in ESI$^-$, but most MS responses remained within +/- 20% (SI 2 Figure S2a), corresponding to the lowest SD between molecular descriptor weights (SI 1 Figure S4b). Mostly increased MS responses were observed in APCI$^-$ with a strong dependence on analyte properties (SI 2 Figure S2a) corresponding with higher changes in molecular descriptor weights (SI 1 Figure S4). Results from the negative mode were similar to the positive mode with a high correlation of molecular descriptor rankings for ESI$^-$ (R^2 of 0.70) and total change of ionization processes in APCI$^-$ (R^2 of 0.33). The use of 1 mmol/L H_2O in ESI$^-$ resulted in a more pronounced negative effect of RPSA, corresponding to the worse ionization of molecules with a higher amount of partially charged surface

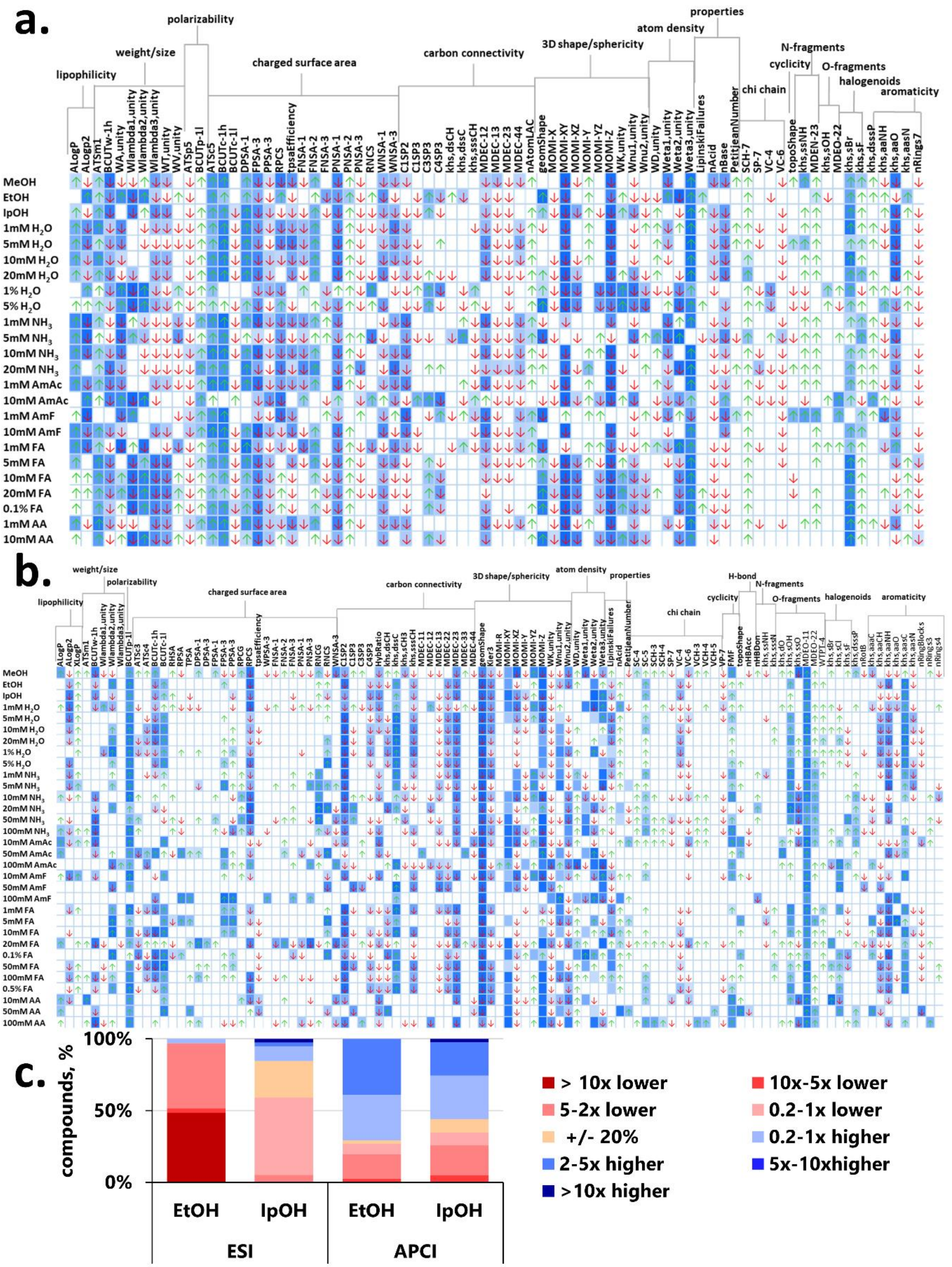

SI 2 Figure S1. Key molecular descriptors affecting ionization in ESI- (a) and APCI- (b) when using MeOH as an organic modifier. The shade of blue in the heatmaps corresponds to a ranking of the molecular descriptor for each makeup solvent composition (ranking 1 = the highest absolute ANN-assigned weight = the darkest blue). ↑- increasing effect on ionization, ↓-decreasing effect on ionization. (c) The effect of ethanol (EtOH) and isopropanol (IpOH) as makeup solvents on MS responses compared to MS responses obtained using MeOH.

area compared to molecules with no surface area with partial charges. Compounds with high total negative charge (low RNCS) were ionized better than molecules with negative charge localized on a small part of the surface using 1 mmol/L H_2O in ESI$^-$. The negative effect of acidic groups (nAcid) and polar surface area (TPSA) was even higher when using H_2O in APCI$^-$, compared to MeOH. Furthermore, low-charged molecules (BCUTc-1l) ionized significantly more efficiently.

The increase in H_2O concentration significantly affected the ionization mechanism in a negative mode, as confirmed by the low correlation coefficients between the weights assigned at different concentrations (SI 1 Table S3). The increase up to 5 mmol/L (SI 2 Figure S2d) had a positive effect on the ionization of over 80% of the compounds in ESI$^-$, but higher concentrations, especially above 20 mmol/L , were detrimental to the ionization of most compounds. In APCI$^-$, H_2O concentration above 1 mmol/L resulted in decreased ionization of most compounds.

Similar to the positive mode, the effect of lipophilicity and molecular weight was dependent on the H_2O concentration, as shown by changing the weights of AlogP2, BCUTw-1h, and Wlambda. Spherical compounds were more easily ionized in ESI$^-$ using 5% H_2O. The least negative effect of sphericity on ionization in APCI$^-$ was observed with 1 mmol/L H_2O. The effect of atom distribution along all three principal component axes (Weta1-3) was important using low H_2O concentrations in APCI$^-$, whereas they did not affect ionization efficiency with higher concentrations of H_2O.

Acids. A strong dependence on analyte properties was observed in ESI$^-$ and APCI$^-$ using 1 mmol/L FA compared to MeOH as both increased and decreased MS responses were determined (SI 2 Figure S2a). Higher SD between molecular descriptor weights (SI 1 Figure S4) confirmed this behavior.

The makeup solvent with 1 mmol/L FA changed the ionization behavior in both ionization sources to a high degree, with R^2 0.4 for ESI$^-$ and 0.2 for APCI$^-$. The main difference between 1 mmol/L FA and MeOH in ESI$^-$ was observed in the effect of the 3D shape of the molecule (SI 2 Figure S1a). The negative weight of geomShape shows preferential ionization of non-spherical compounds, further confirmed by the negative weight of MOMI-Z and the positive weight of MOMI-YZ. The importance of molecular size and distribution along the principal axes was confirmed by Wlambda1, Wlambda2, and Weta2 being among the 20 most influential descriptors using 1 mmol/L FA. 1 mmol/L FA attenuated the negative effect of basic functional groups (nBase), secondary amines (khs.ssNH), and positive surface area (RPCS). Thus, for the ionization of basic compounds in ESI$^-$, 1 mmol/L FA should be used instead of pure MeOH, which seemed to be beneficial for the ionization of compounds with covalently bonded bromine (khs.sBr). The use of 1 mM FA in APCI$^-$ mitigated the negative effect of high MOMI descriptors, secondary oxygens (khs.ssO), and molecular weights (BCUTw-1h). Spherical compounds had weaker ionization using 1 mmol/L FA (negative weight of geomShape), while compounds with low charge (BCUTc-1l), covalently bonded chlorine (khs.sCl), hydroxy groups (khs.sOH), and not following Lipinski's rules were ionized more efficiently. Branching of the molecule with 4-bonded carbon (khs.dssC, khs.aasC) was advantageous in contrast to carbons with bonded hydrogens, which decrease ionization (khs.aaCH, C1SP2).

Mostly lower MS responses were observed at higher FA concentrations in ESI$^-$, while both increased and decreased ionization were observed in APCI$^-$. Similar to ESI$^+$, a high correlation of weights assigned to molecular descriptors using varying FA concentrations ($R^2 > 0.9$) was observed in ESI$^-$. Increasing FA concentration mainly affected the MOMI and geomShape descriptors suggesting that the main effect was related to the drop formation and stability. A significantly smaller effect of the molecular 3D shape was observed for 1 mmol/L AA. Thus, its use may be beneficial when the ionization of compounds with different 3D shapes is required. Varying FA concentrations resulted in more significant changes in APCI$^-$. The ANN-assigned weights correlated with R^2 up to 0.6-0.8. The increasing concentration of FA mainly affected the molecular descriptors related to the 3D shape of the molecule with a similar U-trend as in APCI$^+$. AA at higher concentrations had a more important effect on the ionization and MS responses, because the charge state of the molecule (FNSA, PNSA, WNSA, TPSA) and the chi chain and valence electrons (SC-4, SC-5, SCH-3, VCH-3, VCH-4) affected the ionization to a greater extent than using MeOH and/or FA (SI 2 Figure S1a).

Ammonia. Similar to ESI$^+$, the addition of 1 mmol/L NH_3 to the makeup solvent was critical also in ESI$^-$ as it again increased MS responses for over 90% of analytes (SI 2 Figure S2a). Most MS responses remained comparable to those obtained with pure MeOH in APCI$^-$ (SI 2 Figure S2a). However, for some compounds, a strong increase/decrease in MS responses was observed in APCI$^-$ based on the analyte properties, as also indicated by the higher SD between molecular descriptor weights compared to ESI$^-$ (SI 1 Figure S4).

In ESI$^-$, 1 mmol/L NH_3 increased the negative effect of FNSA-1. Thus, compounds with a localized negative charge were ionized more efficiently than molecules with a higher partial negative surface area. The negative effect of molecular weight (BCUTw-1h) was attenuated. The more efficient ionization of compounds with strong lipophilic properties was observed (AlogP, ALogP2) (SI 2 Figure S1a). The main parameters increasing ionization remained similar to pure MeOH, including high charge (BCUTc-1h, ATSc5, DPSA-1, FNSA-2) and low polarizability (BCUTp-1l). Similar to ESI$^+$, 1mM NH_3 is also recommended as makeup solvent in ESI$^-$ as higher concentrations decreased MS responses for most analytes (SI 2 Figure S2d). Although the molecular descriptor weights from different NH_3 concentrations correlated with $R^2 > 0.7$ (SI 2 Figure S2b), the effect of several molecular descriptors changed, including AlogP2, MOMI-Z, geomShape, and Weta (SI 2 Figure S1a). This again suggests that a change in the evaporation process, droplet formation, and stability are probable. Subsequently, the high abundance of NH_3 is detrimental to the ionization of analytes due to competition for charge, as it is likely already in the NH_4^+ state and cannot act as an H-bond acceptor. The change from 1 mmol/L NH_3 to 1 mmol/L AmAc resulted in slightly decreased MS responses for most compounds. However, the effect of 1 mmol/L AmF was more dependent on the physicochemical properties of the analytes. The effect of Wlambda descriptors decreased using 1 mmol/L AmAc, but overall, the same key molecular descriptors were affecting the ionization as in the case of 1mM NH_3. The use of 1 mmol/L AmF reduced the effect of surface area with negative partial charge (WNSA, PNSA) and 3D shape (MOMI-XY, MOMI-Z). The influence of carbon connectivity/ hybridization and amine groups increased (khs.ssNH, MDEN-23).

The negative effect of several descriptors identified as key descriptors using MeOH as makeup solvent was attenuated by 1 mmol/L NH_3 in APCI$^-$ (SI 2 Figure S1b), including 3D-shape-related geomShape, MOMI-XZ, MOMI-Z, and MOMI-XY, molecular weight (BCUTw-1h), and surface area with partial negative charge (WNSA-3). The importance of atom connectivity increased as khs.sOH, khs.dssC, khs.ssNH, khs.aasC, and khs.aasN ranked significantly higher. Whereas the presence of hydroxy groups and quaternary carbons increased MS responses; aromatic amines decreased ionization. Compounds not conforming to Lipinski's rules were ionized more efficiently. MS responses of most compounds decreased with increasing concentration of NH_3 in APCI$^-$ (SI 2 Figure S2e), mainly affecting the molecular descriptors BCUTw-1h, RNCS, MOMI, and geomShape. A comparison of 10 mmol/L NH_3 vs. 10 mmol/L AmF vs. 10 mmol/L AmAc showed that the two ammonium salts had a contrary effect on MS responses. While two-thirds of the compounds showed higher MS responses using AmAc compared to NH_3, only one-third did so in the case of AmF. The use of ammonium salts improved the ionization of lipophilic compounds (AlogP, XLogP), but cannot be recommended for the ionization of compounds with high ALogP2. Furthermore, the charged surface area (RPCG, RPCS, RNCG) did not affect the ionization using salts compared to NH_3. Contrarily, the positive effect of chlorine substitution was seen using AmF and AmAc.

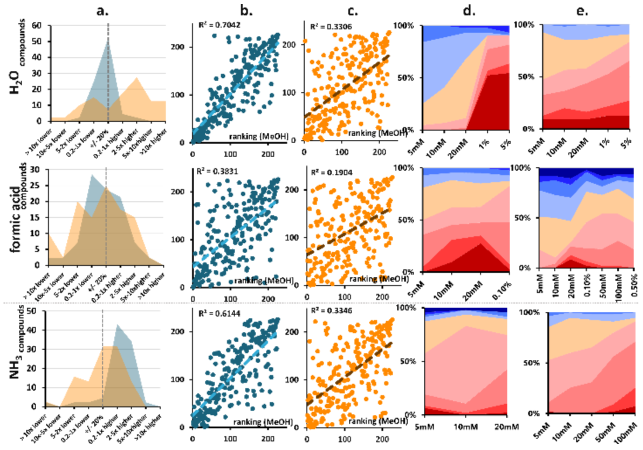

SI 2 Figure S2. Effect of 1 mmol/L additive in makeup solvent compared to MeOH as makeup solvent on MS responses (a) and a comparison of rankings of molecular descriptors based on ANN-assigned weights (b, c) for ESI$^-$ (blue) and APCI$^-$ (orange). Effect of increasing additive concentration on MS responses in ESI$^-$ (d) and APCI$^-$ (e) compared to MS responses obtained using 1 mmol/L additive in methanol. Over 10-fold lower (dark red), 10-5-fold lower (red), 5-2-fold lower (dark pink), 0.2-1-fold lower (pink), within +/- 20% (orange), 0.2-1-fold higher (the lightest blue), 2-5-fold higher (light blue), 5-10-fold higher (blue), over 10-fold higher (dark blue). All results were obtained using MeOH as the organic modifier.

Elucidation of the ionization process in SFC-MS using 10 mmol/L ammonia in methanol as organic modifier. ***Negative ionization mode.*** *Ionization mechanisms using methanol as makeup solvent.* According to their ANN-assigned weights, partially charged molecules with higher TPSA values are ionized more efficiently in both tested ionization sources. The value of the positive and/or negative charge plays an important role here. DPSA-1 and FPSA-3 related to the positive partial charge decreased ionization in ESI$^-$, while they increased ionization in APCI$^-$ suggesting increased ionization of molecules with positive surface areas. The small negative partial charge on the molecular surface was beneficial in ESI$^-$, as shown by the positive weights of the charge-weighted descriptors FNSA-3, PNSA-3, and WNSA-3. The presence of primary oxygen atoms increased ionization in ESI$^-$ (khs.sOH, MDEO-11), which correlated with the positive effect of H-bond donor groups (nHBDon). Secondary and tertiary amines (khs.ssNH, khs.sssN) could also increase ionization, but a larger molecular distance edge between these groups was beneficial (MDEN-23, MDEN-33). Overall, a low number of basic functional groups was advantageous in ESI$^-$ (SI 2 Figure S3a). Secondary carbons with double bonds increased ionization (C2SP2, MDEC-12) whereas primary carbons (C1SP23, C1SP3, MDEC-11) had decreasing effect (SI 2 Figure S3a). The importance of the carbon hybridization state was further confirmed by the HybRatio descriptor, i.e., a ratio of sp^3 carbons to sp^2 carbons. In APCI$^-$, the strongest effect was observed for oxygens. Secondary oxygens with two single bonds (khs.ssO) suppressed ionization while ketone groups (khs.dO) and aromatically bonded oxygens (khs.aaO) enhanced ionization. A small distance edge between oxygen groups was favorable for ionization (MDEO-11, MDEO-12, MDEO-22). Molecular size expressed as Wlambda1 and Wlambda2 also affected the ionization in APCI$^-$, which further increased with low polarizability (BCUTp-1l) and high values of LipinskiFailure (SI 2 Figure S3b).

The effect of alcohol type. Similar changes based on alcohol type in molecular descriptor weights expressed as SD were observed in ESI and APCI (SI 1 Figure S3d). A mostly negative effect on ionization was observed with EtOH and IpOH in ESI$^-$, with IpOH providing the same and/or slightly increased MS responses compared to MeOH in APCI$^-$ (SI 2 Figure S3c). The importance of secondary and tertiary nitrogens (MDEN-23) and polarizability (BCUTp-1l) increased in ESI$^-$ (Figure 8a) with EtOH makeup solvent. The number of quaternary carbons (C4SP3) and the charge state of the molecule (BCUTc-1h) mainly affected the ionization in ESI$^-$ using IpOH. The use of EtOH and especially IpOH in APCI$^-$ mitigated the positive effect of LipinskiFailure and ketone oxygens (khs.dO) on the ionization efficiency (SI 2 Figure S3b) but increased the negative effect of RPCS and sphericity (geomShape).

The effect of additive. Water. The use of 1 mol/L H_2O instead of MeOH enhanced ionization in ESI$^-$ (SI 2 Figure S4a), whereas both decreased and increased responses were observed in APCI$^-$. High SD between the molecular descriptor weights were calculated for both ionization sources (SI 1 Figure S4d).

Opposite to ESI$^+$, the addition of 1 mol/L H_2O to the makeup solvent completely changed the ionization mechanism in ESI$^-$ (R^2 of 0.29, SI 2 Figure S4b). While the negative surface area (FNSA-3) and the large distance edge between primary and secondary oxygens (MDEO-12) increased the ionization efficiency using pure MeOH, the relative positive charge (RPCG) and lipophilicity played a more important role using 1 mol/L H_2O. Compounds with higher logP (AlogP) ionized more efficiently. However, compounds with high AlogP2

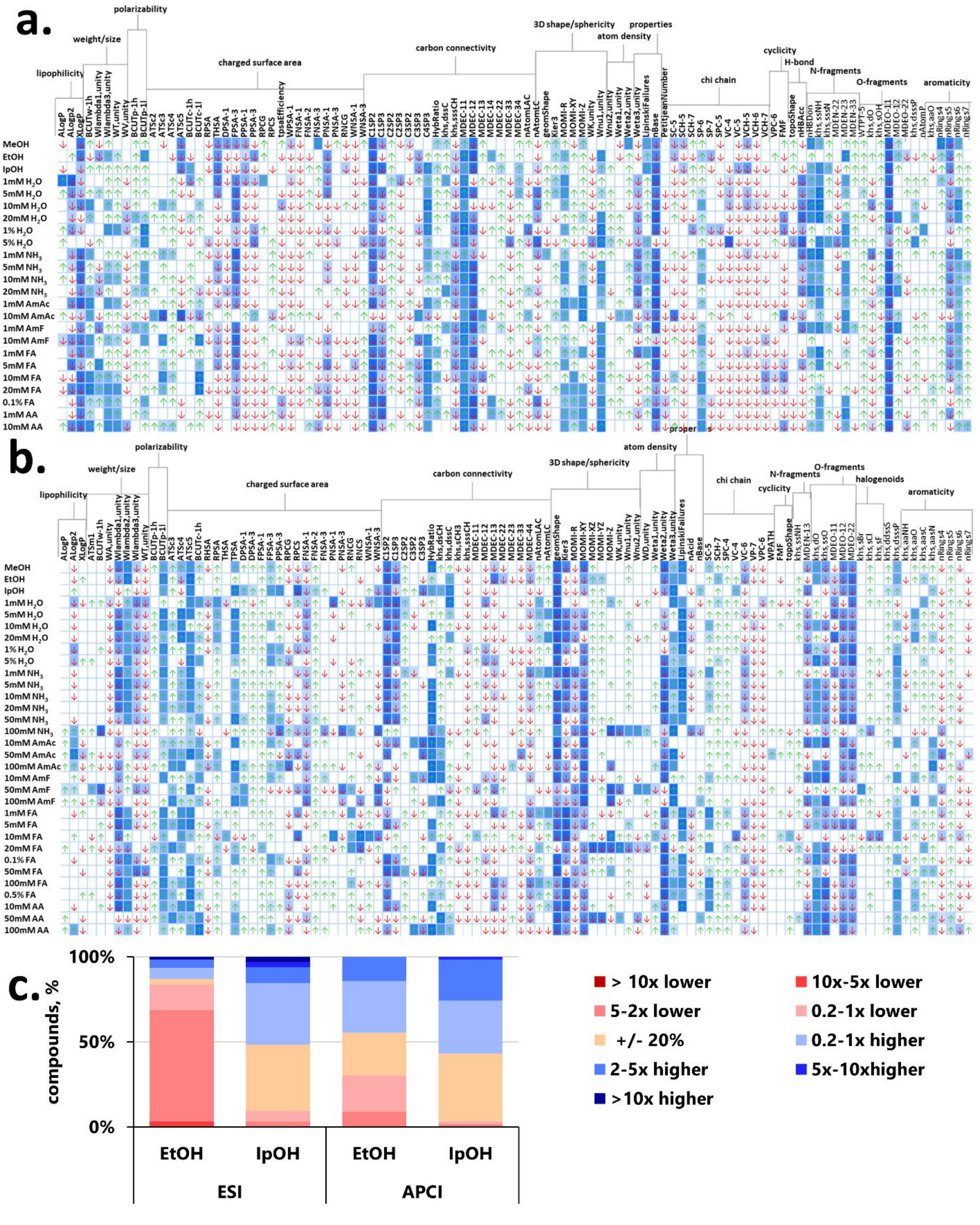

SI 2 Figure S3. Key molecular descriptors affecting ionization in ESI- (a) and APCI- (b) when using 10 mmol/L NH_3 in MeOH as organic modifier. The shade of blue in the heatmaps corresponds to the ranking of the molecular descriptor for each makeup solvent composition (ranking 1 = the highest absolute ANN-assigned weight = the darkest blue). ↑- increasing effect on ionization, ↓-decreasing effect on ionization. (c) The effect of ethanol (EtOH) and isopropanol (IpOH) as makeup solvents on MS responses compared to MS responses obtained using MeOH.

ionized with difficulty. This shows that the branching of the molecule and intermolecular interactions need to be taken into account. The decrease in ionization efficiency was also caused by a high number of H-bond acceptors (nHBAcc), corresponding to a reluctance to form a deprotonated molecule. Completely different ionization processes were also observed in APCI$^-$ (SI 2 Figure S4c). Sphericity, i.e., geomShape, remained the most influential molecular descriptor with significantly increased weight after the water addition. This effect was confirmed by the high positive weight assigned to the MOMI-Z, i.e., corresponding to spherical molecules. Furthermore, the ionization was strongly affected by the partial charge on the surface area. Molecules with the majority of their surface area covered by partial charge (RPSA) were more easily ionized in APCI$^-$, which was confirmed by the negative weights assigned by ANN to THSA. A positive partial charge was more favorable for the ionization as the ranking of molecular descriptors related to positive surface area (RPCS and DPSA-1) increased. Molecular descriptors related to the negative surface area, WNSA-1, WNSA-3, and PNSA-1, became the most influential descriptors for decreasing ionization.

The use of higher concentrations of H_2O in makeup solvent is not recommended in ESI$^-$ as lower MS responses were observed for most compounds (SI 2 Figure S4d). Changes in APCI$^-$ were smaller and strongly dependent on the analyte properties (SI 2 Figure S4e). The effect of polarizability (BCUTp-1l) increased with increasing concentration of H_2O in ESI$^-$ (SI 2 Figure S3a). Conversely, an abrupt change in the ionization mechanism was observed in APCI$^-$ between 1 mmol/L H_2O and higher concentrations, corresponding to the PCA, where 1 mmol/L H_2O is outside of the APCI cluster (Figure 1).

Acids. The use of 1 mmol/L FA in MeOH instead of MeOH as makeup solvent was detrimental (SI 2 Figure S4a) for ionization in both ESI$^-$ (R^2 of 0.3) and APCI$^-$ (R^2 of 0.4), respectively (SI 2 Figure S4b,c, SI 1 Figure S4). In ESI$^-$, the negative effect of the high difference between positive and negative surfaces was slightly mitigated (DPSA-1), but molecules with a high surface area covered by positive charge were still less efficiently ionized (FPSA-3), corresponding to the negative weight of nBase (SI 2 Figure S3a). Molecules with a high number of H-bond donor groups and not conforming to Lipinski rules (LipinskiFailure), i.e., high molecular weight (BCUTw-1h) and hydrophilicity (low XlogP), ionized more easily. For APCI$^-$, the low polarizability (BCUTp-1l) and non-compliance with Lipinski´s rules remained the most important descriptors increasing ionization. Molecular sphericity (geomShape) decreased ionizability. Molecules with high relative positive charge surface area (RPCS) had lower ionizability compared to compounds with localized negative charge and low total negative charge (RNCG). Here, the effect of molecular distance edge between primary and secondary oxygens and nitrogens (MDEO-12, MDEO-22, MDEN-13) was slightly mitigated (SI 2 Figure S3b).

Similar MS responses (SI 2 Figure S4d) and ionization behavior (R^2 > 0.6, SI 1 Table S3) were observed with different FA concentrations in ESI$^-$. Thus, 1mM FA can be recommended in SFC-MS method development as increasing FA concentration increases the possibility of competition for charge resulting in decreased MS responses for some compounds (SI 2 Figure S4d). The use of AA correlated closely to FA (R^2 > 0.6). Various concentrations of FA behaved similarly in APCI except for 10 mmol/L, 20 mmol/L, and 0.1%, where different molecular descriptors, such as TPSA and FPSA-3, became more important. Again, careful optimization should be carried out when using APCI$^-$.

Ammonia. Slightly lower MS responses were observed for most compounds in ESI$^-$ when 1 mmol/L NH_3 was used as makeup solvent instead of MeOH. Slightly higher responses were observed in APCI$^-$ (SI 2 Figure S4a). In ESI$^-$, the change from MeOH to 1mM NH_3 completely mitigated the positive effect of FNSA-3, while the negative effect of the presence of H-bond acceptors, basic groups, keto groups, and strong lipophilic and hydrophilic properties became more significant (SI 2 Figure S3a). H-bond donors and -NH- groups (khs.ssNH) in the molecule enhanced ionization. Increasing NH_3 concentration did not significantly affect the ionization as MS responses of most compounds remained within +/- 20% with only a few exceptions. Molecular descriptor weights affected by concentration change included khs.ssNH, MDEO-12, and the 3D shape related descriptors MOMI-Z and geomShape. Overall, the use of ammonia in the makeup solvent is typically not beneficial in ESI$^-$ when using the MeOH+NH_3 modifier.

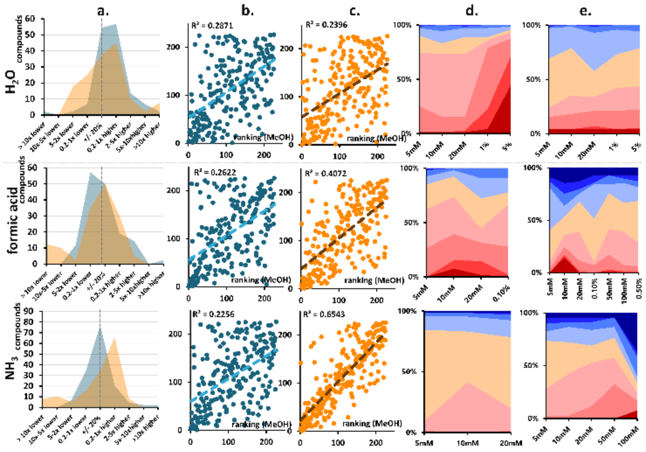

SI 2 Figure S4. Effect of 1 mmol/L additive in makeup solvent compared to MeOH as makeup solvent on MS responses (a) and a comparison of rankings of molecular descriptors based on ANN-assigned weights (b, c) for ESI$^-$ (blue) and APCI$^-$ (orange). Effect of increasing additive concentration on MS responses in ESI$^-$ (d) and APCI$^-$(e) compared to MS responses obtained using 1 mmol/L additive in methanol. Over 10-fold lower (dark red), 10-5-fold lower (red), 5-2-fold lower (dark pink), 0.2-1-fold lower (pink), within +/- 20% (orange), 0.2-1x higher (the lightest blue), 2-5-fold higher (light blue), 5-10-fold higher (blue), over 10-fold higher (dark blue). All results were obtained using 10 mmol/L ammonia in MeOH as the organic modifier.

Similar to APCI$^+$, the change from MeOH to 1 mmol/L NH_3 in MeOH as a makeup solvent had only a minor effect on the ionization behavior with a high correlation, i.e., R^2 of 0.7, of the molecular descriptor weights (SI 2 Figure S4a,c). The effect of positive charge, i.e., PPSA-3, RPCG, decreased, while the carbon connectivity of the molecule became more important, confirmed by high positive weights of nAtomLC related to the long chains, and double bonded carbons (khs.dssC). The benefit of increased NH_3 concentration

was observed only for 100 mmol/L concentration. The weights of molecular descriptors became significantly more pronounced at 100 mmol/L NH_3 compared to 50 mmol/L (SI 2 Figure S3b). The ionization in such concentration level of NH_3 depends on analyte properties related to the charged surface (PPSA-3, PNSA-3, WNSA-3), molecular distribution (Weta), and shape (Wnu, WK, MOMI-Z, geomShape).
